# Trait variations and expression profiling of OsPHT1 gene family at the early growth-stages under phosphorus-limited conditions

**DOI:** 10.1038/s41598-021-92580-7

**Published:** 2021-06-30

**Authors:** Annamalai Anandan, Chidambaranathan Parameswaran, Anumalla Mahender, Amaresh Kumar Nayak, Sampthamprajan Vellaikumar, Cayalvizhi Balasubramaniasai, Jauhar Ali

**Affiliations:** 1 Crop Improvement Division, Indian Council of Agricultural Research (ICAR)-National Rice Research Institute (NRRI), Cuttack, Odisha 753006 India; 2 Rice Breeding Platform, International Rice Research Institute (IRRI), Los Baños, Laguna, 4031 Philippines; 3 Agricultural College and Research Institute, Tamil Nadu Agricultural University, Madurai, 641003 India

**Keywords:** Plant breeding, Plant genetics, Plant stress responses

## Abstract

To better understand the early response of genotypes to limited-phosphorus (P) conditions and the role of the phosphate transporter *OsPHT1* gene family in the presence of *PSTOL1*, it is essential to characterize the level of tolerance in rice under limited-P conditions. In the present experiment, six rice genotypes were studied in three-way interactions [genotype (G) × phosphorus (P) × duration (D)] by comparing them at two instances (14 d and 28 d) under seven different concentrations of P (0.5‒10.0 ppm) in a hydroponic system. Trait differences and interactions of these traits were clearly distinguished among the various P rates. However, aboveground trait expression registered increased growth from 6.0 to 10.0 ppm of P. The major root-attributed traits in 0.5 ppm of P are significantly increased vis-à-vis 10 ppm of P. Analysis of variance displayed a significant difference between the genotypes for *PSTOL1* and *PHT1* expression. In low P, maximum root length with a shoot and root dry weight was observed in a new indigenous accession, IC459373, with higher expression of *PSTOL1* than in Dular and IR64-Pup1 in 0.5 ppm of P at 14 d. Among the 13 *PHT1* genes, *OsPT1*, *OsPT2*, *OsPT6,* and *OsPT13* showed significant upregulation in IC459373, Dular, and IR64-Pup1. These results indicated that studying the expression levels of the *PSTOL1* and *PHT1* gene family at the early growth stages would be helpful in identifying superior donors to improve low-P tolerance and P-use efficiency in rice breeding programs.

## Introduction

Intensive farming and minimal application of phosphate fertilizer are plausible reasons for a substantial decrease in the availability of phosphorus to plants. The heterogeneous nature of soil in both space and time, the formation of a complex with aluminum and iron in acidic pH, and the reactive nature with calcium and magnesium in alkaline-medium result in the unavailability of phosphorus (P) as a nutrient for plants^1^. Upland soils are generally low in available P, and deficiency in the convenient form of P is mounting in most of the cultivable land of India. Because phosphorus is non-renewable in nature, decreased mobility in soil solution and 80% loss in applied P fertilizer increase nutrient deficiency in crops, which ultimately affects grain yield. On the other hand, the rice crop is poor in P-use efficiency (PUE) (~ 25%)^2^. Hence, to optimize the use of available P and to improve PUE, the plant adapts by exhibiting phenotypic plasticity for above- and belowground parts. This adaptive morphogenesis varies among species and genotypes^3^. The role of P is vital for life-sustaining processes such as photosynthesis, respiration, and protein phosphorylation. Further, it has an essential role in nucleic acid, membrane phospholipids, ATP, and NADPH^4, 5^. Changes in morpho-physiological traits such as an increase in the root-to-shoot ratio, accumulation of anthocyanin (pigmented/dark green leaves), changes in the efficiency of photosynthesis and respiration^6^, and an increase in acid phosphates are used by plants to increase the availability of P. These were used as surrogate traits to identify genotypes with low-P tolerance. The root is the chief and hidden organ, hard to separate from the soil and complex to measure manually that intercepts immobile P by altering the root architecture, which is variable in time and space^7^.

Hydroponics is an alternative option to study changes in root architecture by creating significant nutrient stress. Genotypic variations are often reported in response to limited P and changes in root growth^8, 9^. The plasticity of root system architecture is nutrient specific and under systemic control by plant nutritional status^10^. Observed the plasticity of the root system architecture in *Arabidopsis* in response to 12 nutrient deficiencies. The responsiveness of roots and different root parameters varies for each nutrient with the rate of concentration. Such changes reflect that the plant adapts different strategies for each nutrient in a dose-dependent manner. Often, root dry weight and maximum root length were studied to understand the magnitude of the response of genotypes under limited P^9, 11^. However, scarce reports are available on root architecture, with hardly any in-depth analysis of root system architecture under different rates of P. In addition, inter-relating individual root parameters among them and with aboveground traits in different rates of P will give further insights into understanding the changes happening because of P.

In response to nutrient deficiency, physiological and molecular changes lead to modification in morphological traits above and below ground level.. Therefore, understanding the expression of genes related to P transporters and *P starvation tolerance 1(PSTOL1)* in the root would help understand the modulation of root architecture to improve Pi acquisition under limited P. Recently Chiou et al. comprehensively reviewed the molecular mechanism and expression of 26 *OsPHT* genes in specific tissues^12^, that distributed across 11 chromosomes. Among them, 13 *OsPHT* genes of *PHT1* (phosphate transporter) were reported to be expressed in the rhizodermis and cortical cells of rice roots. Of these, *OsPHT1*:1/2/4/6/8/9/10 are principally involved in P uptake. *PHT1* is the phosphate transporter gene family that has a significant role in Pi acquisition, a process driven by plasma membrane H^+^-ATPase^13^. Besides, the identification and introgression of PSI protein kinase encoding the *PSTOL1* gene is a leap forward in the development of rice with low-P tolerance by enhancing early root growth in P-deficient soil^14^. Although modern rice cultivars are high-yielding, they have lower root fractions and are more responsive to nutrients^15, 16^. On the contrary, landraces are less responsive and differ widely in root physiological characteristics, nutrient acquisition ability, and nutrient uptake per unit root length^17^. Based on these facts, we hypothesized the present work in genotypes that have the *PSTOL1* gene, with the following objectives aiming to understand (i) the relative growth rate of traits under different concentrations of P, (ii) the threshold rate of P at early growth stages, and (iii) the role of early root vigor in shoot growth under P-deficiency conditions and the importance of the phosphate transporter *OsPHT1* gene family in the presence of *PSTOL1* to comprehend their rate of tolerance under low P. Therefore, perceptive information from our study, through information on the factors responsible for genotypic variation related to early root vigor and changes in specific root traits related to low P, could signify prospects for future crop improvement in rice.

## Results

### ANOVA of morpho-physiological traits at 14 d and 28 d

Twenty-one phenotypic traits were analyzed in six different genotypes having *PSTOL1* (except IR64) under different phosphorus concentrations (0.5‒10.0 ppm) at 14 d and 22 traits at 28 d. The ANOVA for 14 d showed a significant difference between the genotypes, P concentration, and also their interaction for the studied phenotypic traits. The maximum variation among the genotypes was observed for shoot P content (74.57%), followed by maximum root length (39.90%), root surface area (32.12%), total root length (31.75%), and shoot length (31.10%) (Table 1). Similarly, maximum variation for different P concentrations at 14 d was contributed by P content in roots (78.71%), followed by root dry weight (56.53%) and root length per volume (40.25%). The traits stem dry weight (33.46%), average root diameter (32.05%), leaf width (30.60%), and root volume (30.47%) contributed maximum variation for the genotypes and their interaction with P concentration (G × C). In comparison with 14 d, ANOVA of traits at 28 d also showed a significant difference between the genotypes, P concentration, and their interaction (Table 2). Further, traits contributing to maximum variability at 14 d also showed considerable variation at 28 d. However, the projected root area contributed 51.42% variation at 28 d vis-à-vis 27.71% at 14 d between the genotypes. Similarly, leaf width (29.43%) contributed a higher difference at 28 d than at 14 d (5.92%) for different P concentrations. The interaction of G × C at 28 d showed that the number of tillers, shoot dry weight, and root dry weight contributed to higher variation than at 14 d. Even though several traits (18) showed a statistically significant difference at 14 d, three traits (leaf dry weight, shoot dry weight, and average root diameter) were found to show non-significant differences. Similarly, root volume was found to show non-significant differences at 28 d.

**Table 1:** Analysis of variance for various traits across different concentrations of phosphorus at 14 d.

Traits	Grand mean	Genotype (G) MSS	Contribution (%)	Concentration (C) MSS	Contribution (%)	G × C MSS	Contribution (%)
Shoot length	37.40	167.84**	31.10	43.44**	9.66	22.51**	25.03
Max. root length	14.16	193.35**	39.90	137.94**	34.16	5.96 ns	7.38
Leaf length	19.45	55.43**	19.44	30.65**	12.90	9.45 ns	19.89
Leaf width	0.371	0.062**	27.81	0.011*	5.92	0.011**	30.60
Number of leaves	4.94	2.375**	30.06	1.008**	15.31	0.297**	22.55
SPAD	30.04	53.29**	13.01	36.42**	10.67	18.14 ns	26.58
Stem diameter	1.33	0.067 ns	6.02	0.167**	17.93	0.052*	27.74
Leaf dry weight	0.050	0.003 ns	4.96	0.002 ns	4.75	0.002 ns	24.05
Stem dry weight	0.026	0.0002**	15.57	0.0001 ns	5.69	0.0001**	33.46
Shoot dry weight	0.076	0.004 ns	5.95	0.003 ns	5.53	0.003 ns	25.94
Root dry weight	0.014	0.0001**	8.88	0.0006**	56.53	0.00004**	16.31
Root-to-shoot ratio	0.382	0.095**	27.17	0.114**	39.15	0.006 ns	9.77
Leaf area	5.44	11.64**	15.49	7.24**	11.56	3.22 ns	25.74
Total root length	52.45	2769.86**	31.75	2344.55**	32.25	280.93*	19.32
Projected root area	11.33	82.74**	27.71	63.48**	25.52	9.75 ns	19.59
Root surface area	12.30	38.11**	32.12	25.55**	25.84	4.45 ns	22.49
Average root diameter	0.415	0.0130 ns	7.02	0.0144 ns	9.32	0.0099 ns	32.05
Root length per volume	159.74	13,812.60**	20.48	22,628.30**	40.25	2515.84*	22.38
Root volume	0.255	0.1322 ns	7.59	0.2302*	15.87	0.0884 ns	30.47
Shoot P content	0.90	0.0769**	74.57	0.0178**	20.76	0.0005*	2.74
Root P content	0.33	0.0014**	10.83	0.0084**	78.71	0.0001 ns	3.56

*Significant at 0.05 level of probability.

**Significant at 0.01 level of probability.

**Table 2:** Analysis of variance for various traits across different concentrations of phosphorus at 28 d.

Traits	Mean	Genotype (G) MSS	Contribution %	Concentration (C) MSS	Contribution %	G × C MSS	Contribution %
Shoot length	57.65	652.35**	26.78	233.41**	11.50	116.898**	28.79
Max. root length	17.52	390.15**	50.29	101.66**	15.72	19.948**	15.43
Leaf length	31.34	305.24**	21.66	186.12**	15.85	60.701**	25.84
Leaf width	0.51	0.0144**	7.91	0.0442**	29.43	0.0089**	33.46
Number of leaves	6.79	10.08**	32.62	3.124**	12.12	1.159*	22.49
Number of tillers	1.05	0.274**	20.77	0.0926**	8.40	0.0894**	40.58
SPAD	34.13	58.80**	22.59	9.625 ns	4.44	13.089**	30.18
Stem diameter	1.80	0.218 ns	3.22	1.491**	26.43	0.239 ns	21.17
Leaf dry weight	0.13	0.0132**	20.79	0.0054**	10.27	0.0032**	30.30
Stem dry weight	0.074	0.0029**	11.66	0.0010 ns	4.68	0.0014**	34.75
Shoot dry weight	0.209	0.0279**	21.36	0.0100**	9.23	0.0083**	38.36
Root dry weight	0.038	0.0012**	11.64	0.0020**	22.63	0.0006**	35.74
Root-to-shoot ratio	0.30	0.0778**	38.34	0.0625**	36.93	0.0032*	9.39
Leaf area	12.23	40.047**	9.49	87.132**	24.77	21.832**	31.03
Total root length	120.25	43,780.20**	43.70	13,871.20**	16.61	4088.69**	24.49
Projected root area	15.49	222.55**	51.42	37.56**	10.41	14.28 ns	19.80
Root surface area	17.08	225.76**	52.60	62.91**	17.59	11.29 ns	15.78
Average root diameter	0.408	0.00752 ns	5.52	0.03579**	31.52	0.00688 ns	30.27
Root length per volume	400.83	217,780.00**	50.68	19,186.70 ns	5.36	17,959.50*	25.08
Root volume	0.67	1.55175 ns	12.65	1.14796 ns	11.23	0.62349 ns	30.49
Shoot P content	0.95	0.0354	63.803	0.0119**	25.76	0.0006**	6.531
Root P content	0.39	0.0025	12.268	0.0137**	80.24	0.0002**	4.761

*Significant at 0.05 level of probability.

**Significant at 0.01 level of probability.

### Biplot analysis of P concentration and traits at 14 d and 28 d

**Figure 1:**
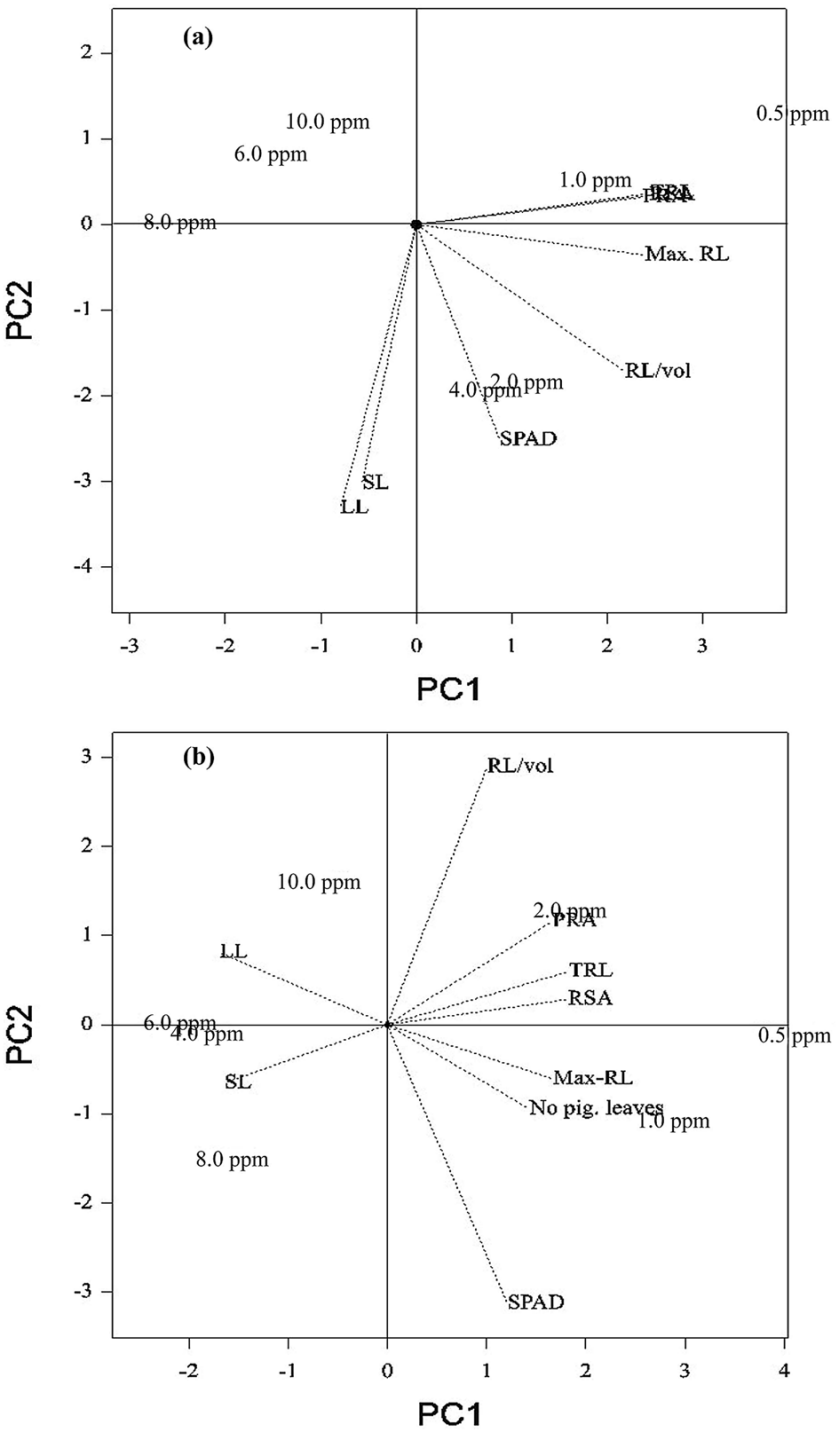
Concentration-by-trait biplot based on the variance exhibited by selected traits under different concentrations of phosphorus explained by two principal component axes: (**a**) 14 days after sowing and (**b**) 28 days after sowing.

In ANOVA, 18 out of 21 traits showed a statistically significant difference for P concentration. Hence, biplot analysis was carried out to identify the variation between the traits for P concentration. At 14 d, the first component (PC1) contributed 97.08% variation for P concentration and traits (Fig. S1). Also, PC1 differentiated lower P concentrations (0.5 ppm, 1.0 ppm, and 2.0 ppm) from the four higher P concentrations. Further, eight highly variable traits (shoot length, leaf length, SPAD, maximum root length, root length/volume, projected root area, root surface area, and total root length) were selected to understand the relationship of traits with P concentration. In the biplot analysis, the eight selected traits contributed 97.08% and 2.75% variation in PC1 and PC2, respectively. The trait-concentration relation analysis showed that the traits projected root area, root surface area, and total root length were grouped under 0.5 and 1.0 ppm of P concentration (Fig. 1a). Leaf SPAD values, root length/volume, and maximum root length were grouped under 2.0 and 4.0 ppm of P concentration. However, shoot and leaf length were found to have the opposite relationship to the traits mentioned above for 0.5‒4.0 ppm of P concentration. Biplot analysis comprising 22 traits in different P concentrations at 28 d also differentiated low-P concentration from high-P concentration (4.0‒10.0 ppm). Further, biplot analysis for selected highly variable traits showed seven traits (SPAD, number of pigmented leaves, maximum root length, root surface area, total root length, root length per volume, and projected root area) associated with 0.5, 1.0, and 2.0 ppm of P concentration (Fig. 1b). Thus, the grouping of traits with different P concentrations were found to be similar at 14 d and 28 d in our analysis. However, 4.0 ppm of P concentration was differentially grouped with low-P concentration (0.5, 1.0, and 2.0 ppm) and high-P concentration (6.0, 8.0, and 10.0 ppm) at 14 d and 28 d, respectively.

Further, 4.0 ppm of P concentration was considered as threshold P concentration, and differential trait response was analyzed for 14 d and 28 d. All the traits except leaf dry weight (14%), dry stem weight (8%), shoot dry weight (12%), and P content of shoot (8%) and root (12%) exhibited a significant percentage decrease from 6.0 to 10.0 ppm of P concentration vis-à-vis 4.0 ppm at 14 d. Two traits (maximum root length and the ratio of root-to-shoot dry weight) had the maximum decrease of 22% and 33%, respectively, at 8.0 ppm relative to 4.0 ppm. At 28 d, leaf length (6%), projected root area (5%), root-to-shoot ratio (3%), maximum root length (2%), SPAD (2%), leaf area (1%), and root surface area (1%) measured in 6.0 to 10.0 ppm expressed a decrease in growth vis-à-vis 4.0 ppm. Conversely, root length per volume (15%), root volume (13%), and tiller number (11%) attained maximum growth from 6.0 to 10.0 ppm compared to the same traits at 4.0 ppm. Nonetheless, the significant increase in root length up to 1.43-fold at 14 d and average root diameter, maximum root length, root dry weight, total root length, and root volume by 1.25, 1.38, 1.57, 1.60, and 1.67 times in 0.5 ppm, respectively, was observed vis-à-vis the concentration of 10.0 ppm. However, the gain in root parameters under decreased P (0.5 ppm) also had increased percentages of leaf senescence and pigmented leaf numbers, by 2.64- and 2.43-fold, respectively, in 0.5 ppm of P.

### Contribution of the root, shoot, and leaf weight to total plant weight at different concentrations of P

**Figure 2:**
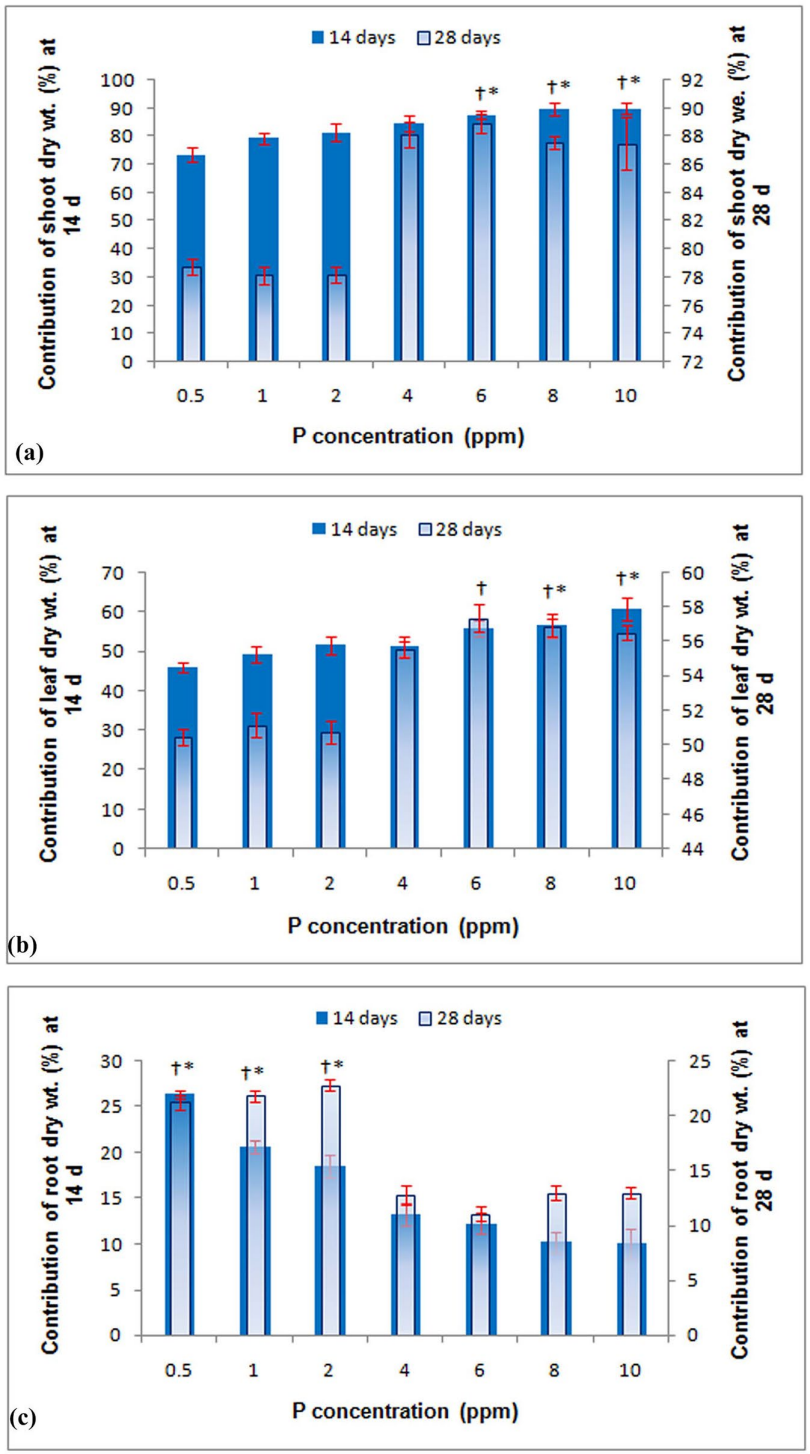
Percentage contribution of shoot/leaf/root to the whole plant under different concentrations of phosphorus in a hydroponic experiment at 14 d and 28 d. Vertical bars represent standard deviation. *p = *0.05* at 14 d, †*p* = *0.05* at 28 d.

In our experiment, six rice genotypes were evaluated under seven different P concentrations to ascertain the contribution of roots, leaves, and shoots to total plant weight. A significant influence was observed for different P concentrations on the root, shoot, and leaf weight (Fig. 2a–c). The trait contribution of roots to total plant dry weight varied from 10.0% to 26.4%, with an average of 14% at both 14 d and 28 d. Specifically, at low-P concentrations (0.5‒2.0 ppm), root contribution to total plant weight was higher (17‒26%) than at higher P concentrations (10‒13%) (Fig. 2c). In contrast, a reverse trend was observed for leaf and shoot tissue contribution to total plant weight (Fig. 2a,b). Shoot tissues contributed to total plant weight at a range of 73% to 90% on both dates of observation. Additionally, higher P concentration (6.0‒10.0 ppm) showed an increased leaf tissue (> 56%) and shoot tissue contribution (> 86%) to total plant weight at both 14 d and 28 d. Further, a proportionate increase in leaf tissue contribution occurred with an increase in P concentration at both dates of observation.

### Effect of different rates of P on the rate of trait development

**Figure 3:**
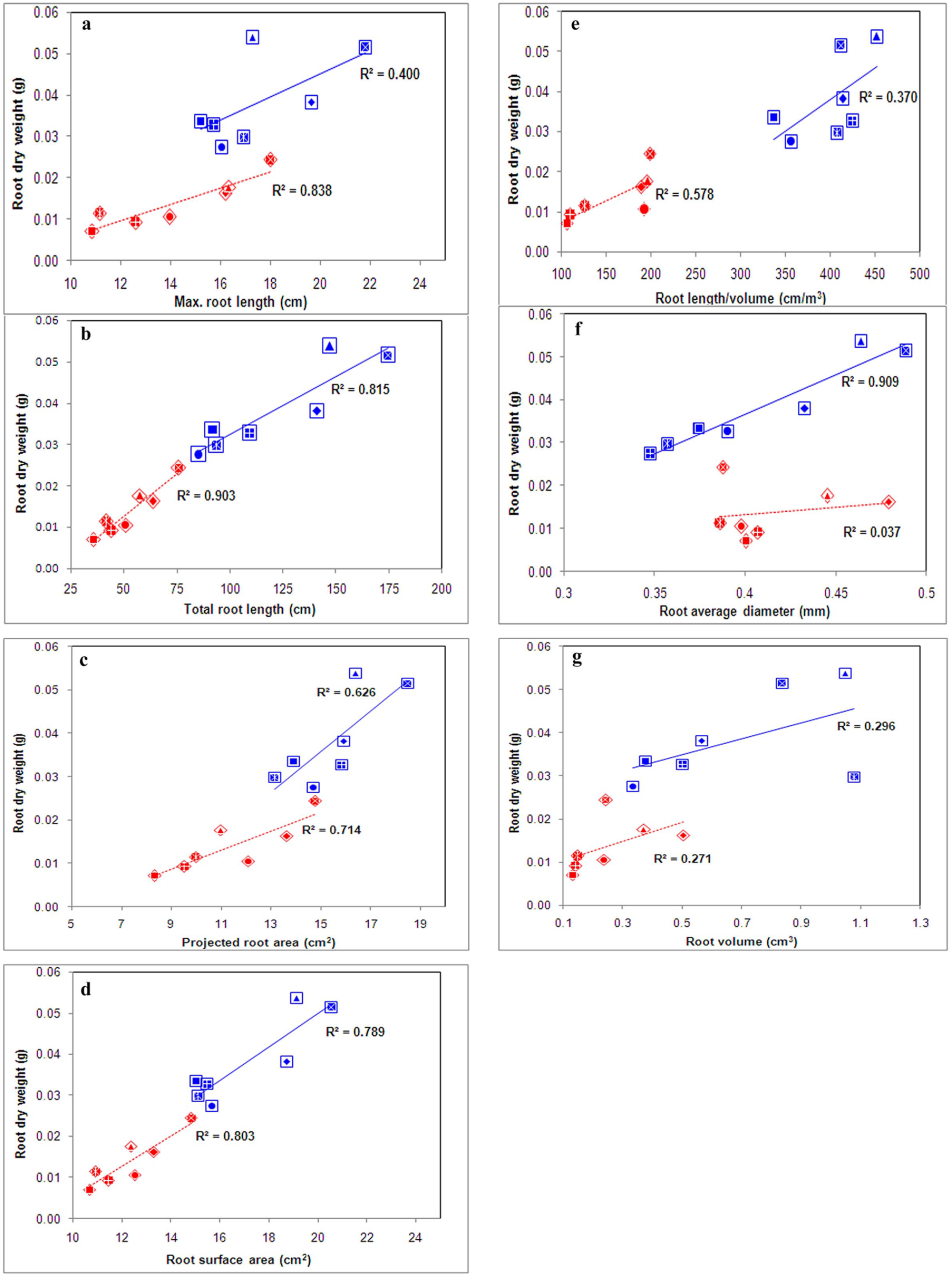
Phenotypic relationship between root dry weight and root parameters across different concentrations of phosphorus under hydroponics at 14 (red = ◊) and 28 (blue = □) days after sowing. ⊠ − 0.5 ppm, ♦ − 1 ppm, ▲ − 2 ppm, ● − 4 ppm,  − 6 ppm, ■ − 8 ppm, and  − 10 ppm.

The rate of change in 21 traits was studied at 14 d and 28 d for differing P concentrations. In low-P concentration, three traits, average root diameter (0.00362), SPAD (0.492), and total root length (7.06), showed an increased rate of change at 28 d relative to 14 d (Table S1). Similarly, 6.0 ppm of P concentration showed an increased rate of development for shoot length (1.86), maximum root length (0.413), leaf length (1.20), leaf area (0.76), stem diameter (0.057), dry leaf weight (0.093), stem dry weight (0.085), shoot dry weight (0.09), and root volume (0.065). The traits leaf width (0.014), leaf number (0.179), root dry weight (0.105), projected root area (0.451), root length per volume (22.52), and root P content (0.005) showed increased trait development with an increase in P (> 8.0 ppm). Conversely, some traits showed decreased growth in differing P concentrations. For example, a decreased growth rate was observed with the aerial portion of plants such as shoot length (1.10), leaf width (0.006), leaf area (0.348), leaf dry weight (0.065), stem dry weight (0.055), stem diameter (0.009), and shoot dry weight (0.061) in < 2.0 ppm of P concentration. Similarly, the growth rate of root-associated traits such as maximum root length (0.248), total root length (2.465), projected root area (0.271), root surface area (0.224), root length per volume (11.68), and root volume (0.008) was found to be affected in 4.0 ppm at 14 d and 28 d. The relative growth rate of root diameter, total root length, and leaf color intensity (SPAD) gained a maximum at low Pi (0.5 and 1.0 ppm). The increase in the concentration of green color and gain in maximum root growth was considered a sign of low P. In addition to these traits, we are reporting that an increase in average root diameter was observed at low Pi at 28 d. This is very well observed from Fig. 3f, where root dry weight had a strong positive association with average root diameter (90%) at 28 d and weak (3%) and non-significant associations across different rates of P (Table 1) at 14 d. This illustrates that an increase in root dry weight under low Pi might be due to an increase in root diameter. Average root diameter was found to increase with a decrease in the availability of P. Therefore, root diameter might be an indicator of the soil strength that affects the acquisition of nutrients. Compared with 10.0 ppm of P, root diameter increased by 25.17% in 0.5 ppm and 1.0 ppm, respectively. The increased diameter might be due to a decrease in the number of root hairs and an increase in aerenchyma. It was observed that the number of dried leaves per plant was 4.02 and 4.74 times higher in 1.0 and 0.5 ppm of Pi, respectively than in 10 ppm.

### Correlation among traits at 14 d and 28 d at varied P concentrations

**Figure 4:**
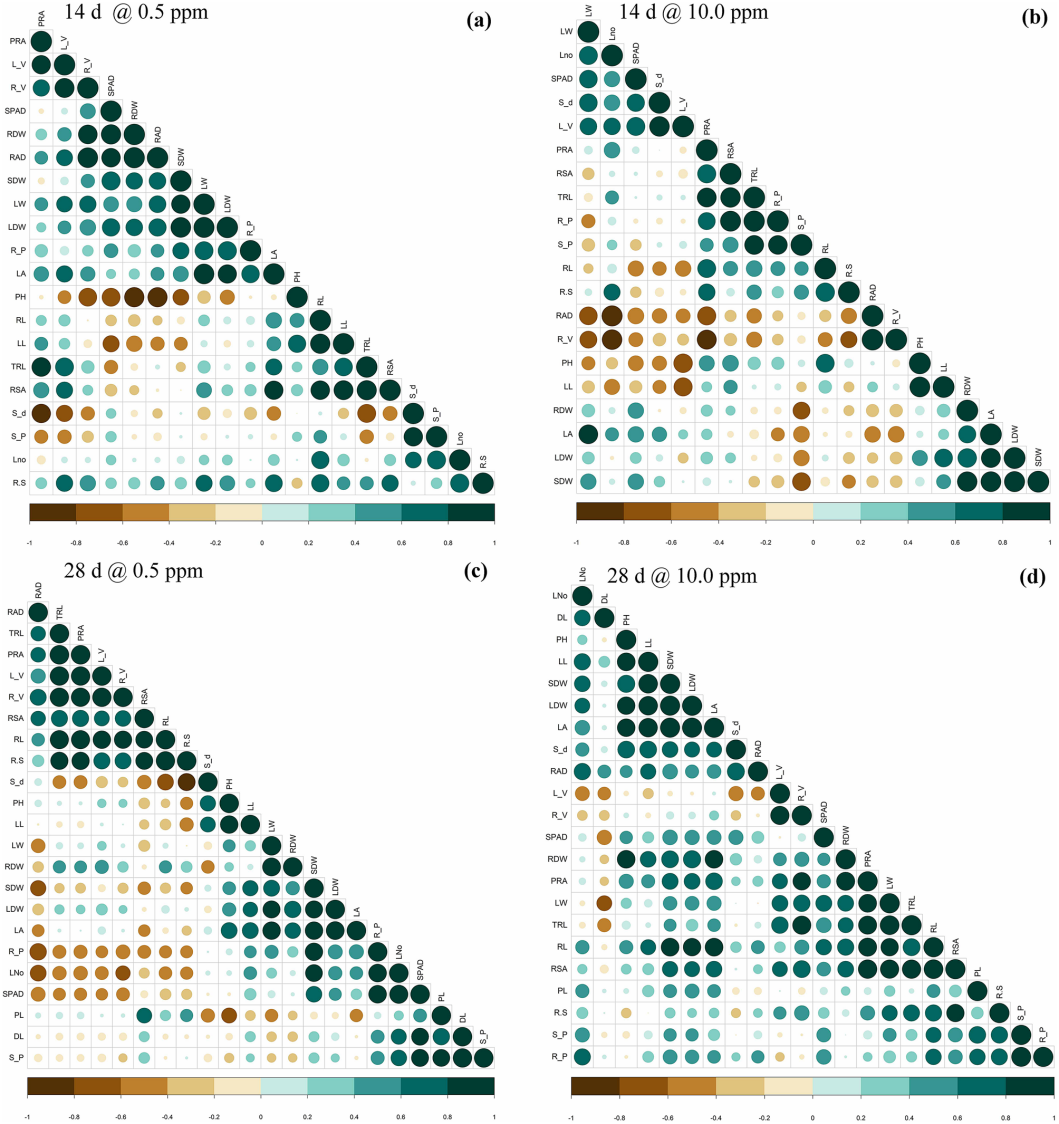
The correlation coefficient between morphological and physiological traits at 14 d and 28 d. a = correlation coefficient at 14 d in 0.5 ppm of P, b = correlation coefficient at 14 d in 10.0 ppm of P, c = correlation coefficient at 28 d in 0.5 ppm of P, and d = correlation coefficient at 28 d in 10.0 ppm of P. DL: number of dry leaves; L_V: root length per volume; LA: leaf area (cm^2^); LDW: leaf dry weight (g); LL: leaf length (cm); LNo: number of leaves; LW: leaf width (cm); PH: shoot length (cm); PL: number of pigmented leaves; PRA: projected root area (cm^2^); R.S: root-to-shoot ratio; R_P: root P content (mg/g); R_V: root volume (cm^3^); RAD: average root diameter (mm); RDW: root dry weight (g); RL: max. root length (cm); RSA: root surface area (cm^2^); S_d: stem diameter (mm); S_P: shoot P content (mg/g); SDW: shoot dry weight (g); SPAD; TRL: total root length (cm).

The relationship among the traits from the different rates of P indicates that low P (0.5‒4.0 ppm) had considerable influence over root growth. Significantly positive correlations were found between total root length and root volume, average root diameter, and leaf dry weight in low P (1.0–4.0 ppm) conditions at both 14 d and 28 d (Fig. 4a–d and Fig. S2). Moreover, root dry weight was significantly and positively associated with root volume, root length per volume, and LDW in 0.5‒4.0 ppm at both dates of observation. Shoot dry weight (2.0‒6.0 ppm), root length (0.5‒4.0 ppm), and leaf area (0.5–6.0 ppm) were found to be positively correlated with root surface area at 14 d. Conversely, under deficient P (0.5–4.0 ppm), average root diameter, shoot length, and leaf number showed a negative association with root dry weight, leaf width, and root volume, respectively, at 14 d and 28 d. The trait projected root area was positively correlated at 14 d and negatively correlated at 28 d with average root diameter under deficient (1.0–4.0 ppm) and sufficient (6.0‒10.0 ppm) P, respectively. These results suggested that the negative association between average root diameter and projected root area exemplified the possibility of changing root architecture in the presence of adequate P by increasing root area by decreasing the diameter to produce more root hairs. Thus, the results showed that root-related traits were found to be strongly associated with P concentration. Further, the number of pigmented leaves was found to be highly significant with root surface area, root length per volume, total root length, and root volume from 0.5 to 4.0 ppm of P. In continuation of this, the number of dried leaves per plant recorded a positive association with the number of pigmented leaves under low P (0.5–4.0 ppm), and they were significantly correlated (0.784**, *p* = 0.01).

### Physiological responses of genotypes for varied P concentration

**Figure 5:**
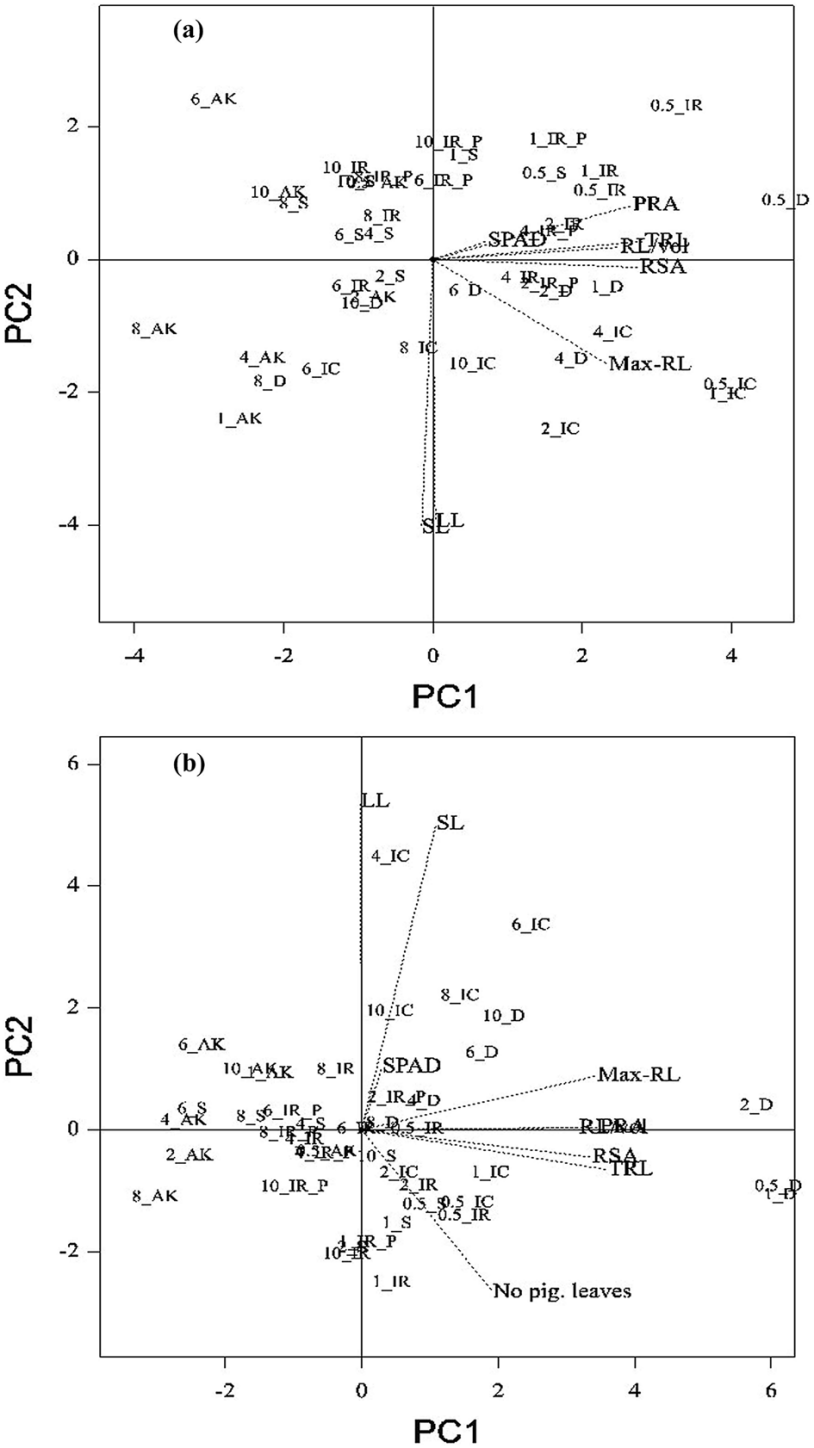
Genotype-by-trait biplot based on the variance exhibited by selected traits under different concentrations of phosphorus explained by two principal component axes: (**a**) 14 days after sowing and (**b**) 28 days after sowing.

Genotypic variation for differing P concentrations was studied through PCA by including 22 traits and 7 different P concentrations. Similar to our previous biplot analysis, nine highly variable traits (shoot length, leaf length, number of pigmented leaves, SPAD, root length, root length per volume, projected root area, root surface area, and total root length) contributed 99% of the variation (Fig. 5). The biplot differentiated low-P concentration (0.5‒4.0 ppm) from high-P concentration at 14 d. Increased root length, maximum projected root area, and root volume per cm were observed in all six genotypes at 0.5–4.0 ppm of P concentration (Fig. 5a). In particular, IC459373 showed maximum root length, while Dular had the highest values for projected root area, total root length, and root length per volume under deficient-P conditions. A similar response of genotypes was also observed at 28 d. In particular, low-P concentrations resulted in an increased number of pigmented leaves in all the genotypes (Fig. 5b). Additionally, higher P concentrations showed increased SPAD values and shoot length irrespective of the genotypes. In particular, genotypes A.Kuruvai (0.5‒10.0 ppm) and Sahbagidhan (2.0‒10.0 ppm) plotted on the extreme left side of the biplot, opposite the concentration (low P) that promoted maximum root growth. Pigmented leaf number in quadrant III was found to be strongly correlated with low P (*p* < 0.05) and all root-related traits (total root length, root surface area, root volume, and length/volume) (Fig. 5b).

### Contribution of the root, shoot, and leaf weight to total plant weight at different rates of P of six genotypes

**Figure 6:**
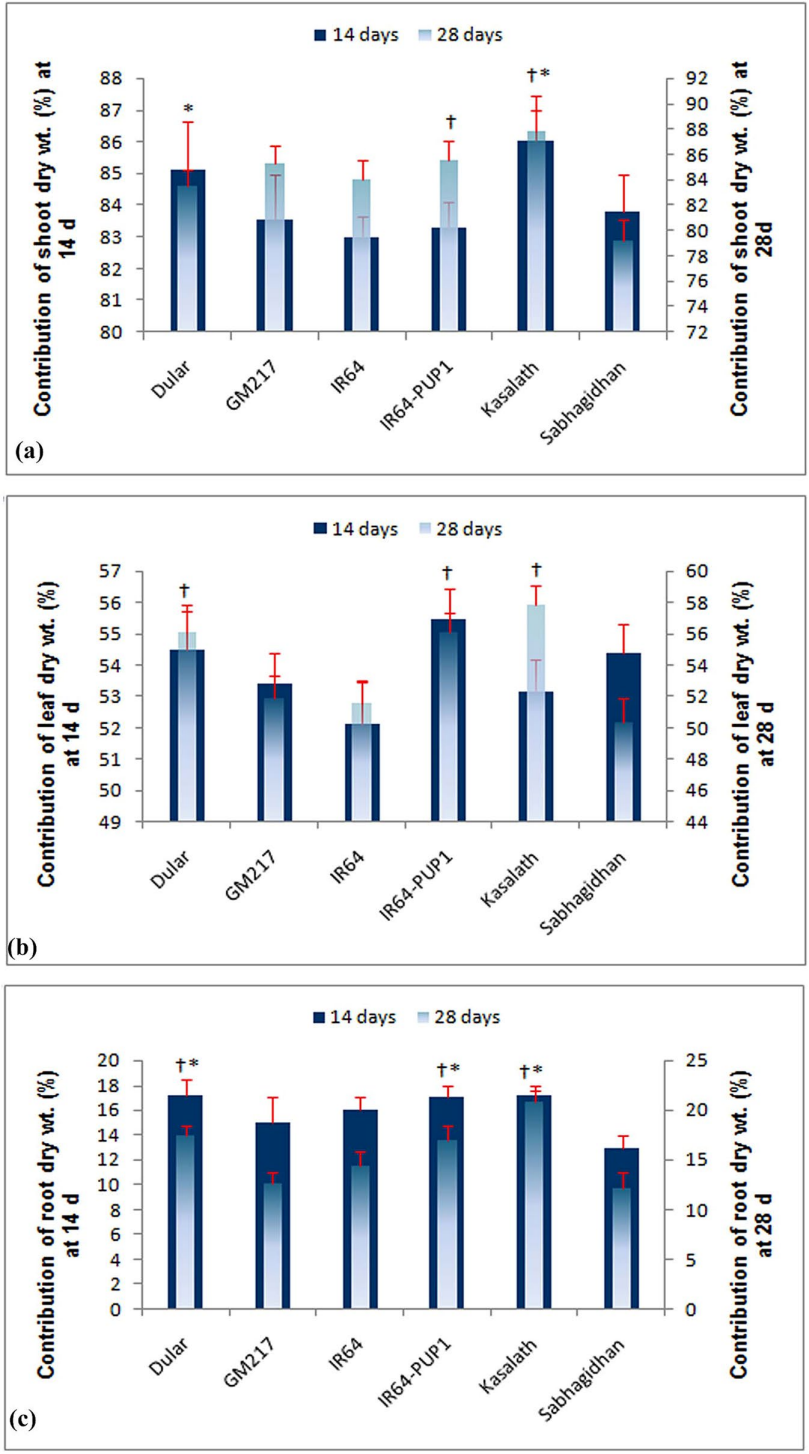
Percentage contribution of shoot/leaf/root dry weight in the total plant weight of genotypes across a different concentration of phosphorus in the hydroponic experiment at 14 d and 28 d. Vertical bars represent standard deviation. *p = 0.05 at 14 d, †*p* = 0.05 at 28 d.

A significant variation was observed among the genotypes in their contribution to total plant weight in different P concentrations at 14 d. Shoot weight contributed 83‒85% of total plant weight at 14 d with genotype Dular (85%), and IC459373 (Kasalath) showed higher shoot weight (86%) than the other genotypes (Fig. 6a–c). However, the contribution of shoot weight to total plant weight increased only for GM127, IR64, and IR64-Pup1 at 28 d, whereas IC459373 showed a similar contribution and Dular and Sahbagidhan showed a decreased contribution at 28 DAS. Similarly, Dular (17%), IC459373 (17%), and IR64-Pup1 (17%) showed a higher contribution of root weight to total plant weight at 14 d. However, only IC459373 (21%) showed an increase in the contribution of root weight to total plant weight at 28 d, whereas all the other genotypes showed a decreased root contribution to total plant weight at 28 d. Similarly, IC459373 showed the highest contribution of leaf weight to total plant weight at 28 d vis-à-vis the other genotypes.

### Variability in the growth rate of genotypes across different P concentrations

Dular and IC459373 showed an increased rate of growth for most of the root parameters in low-P concentrations from 14 to 28 d. Dular showed an increased growth rate for maximum root length (0.5 ppm), total root length (1.0 ppm), root length per volume (1.0 ppm), projected root area (1.0 ppm), and root surface area (2.0 ppm). Similarly, IC459373 showed a higher growth rate for average root diameter (0.5 ppm). Genotypes IR64-Pup1 and Dular showed an increased rate of root volume and root dry weight in 6.0 ppm and 8.0 ppm of P concentration, respectively. Further, IC459373 showed a higher absolute growth rate for shoot length (2.567), leaf length (1.638), and leaf area (0.975) in 6.0 ppm of P concentration. Similarly, IC459373 had a relatively higher increase in leaf width at 8.0 ppm of P, followed by IR64-Pup1 (0.021). Among all the genotypes, A.Kuruvai had a relative increase in dry weight of shoot (0.133), leaf (0.135), and stem (0.129) at 6.0 ppm of P concentration from 14 to 28 d. SPAD value was found to be maximum in Dular at 1.0 ppm (0.705) and minimum in IR64-Pup1 at 10.0 ppm (0.038). Out of six genotypes studied, Sahbagidhan and A.Kuruvai had a minimal growth rate for most of the traits.

### Expression analysis of the *PSTOL1* gene

**Figure 7:**
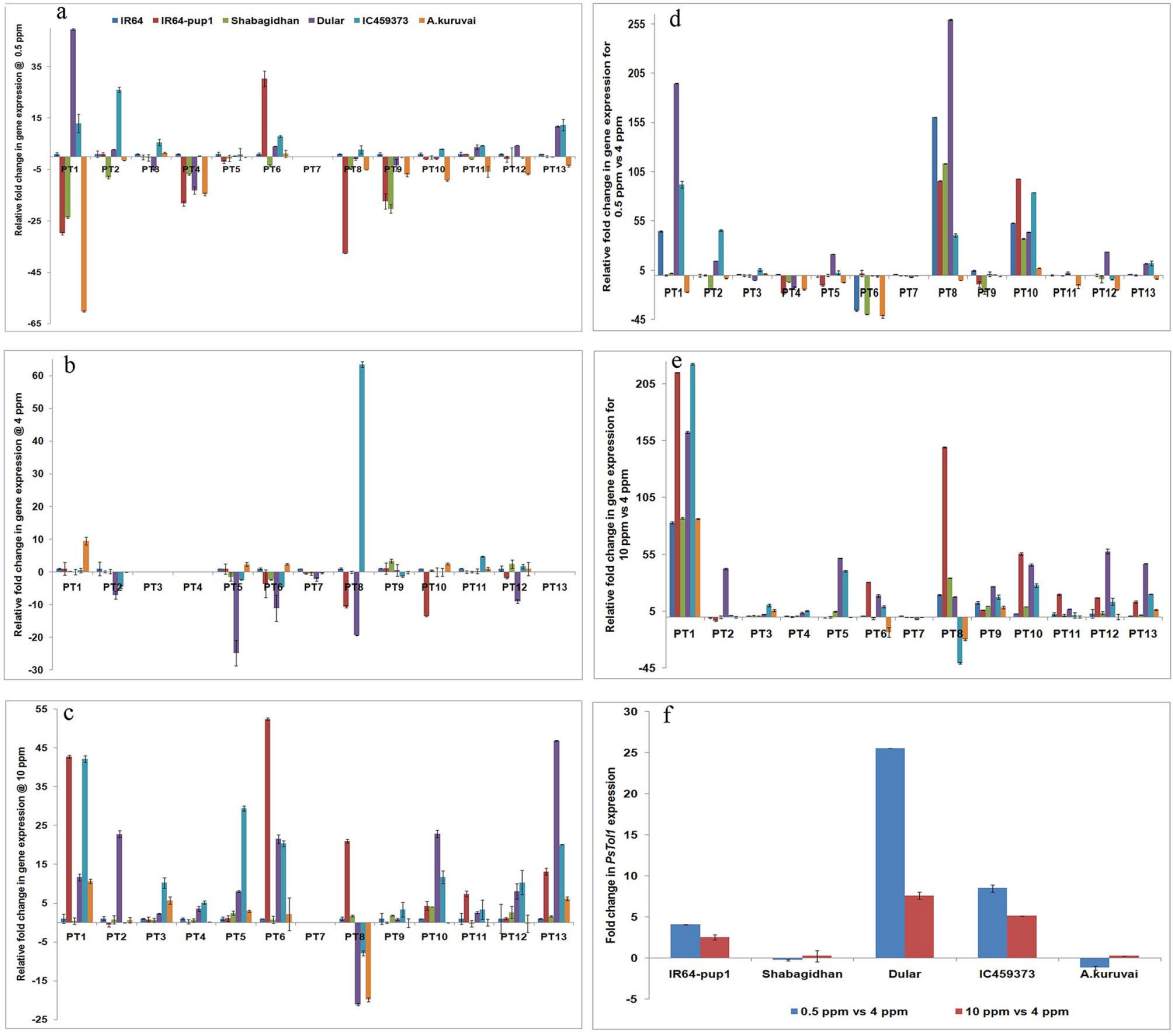
Expression analysis of *PSTOL1* and *OsPT* genes in rice in different concentrations of P. (**a**) Relative fold change in expression of *OsPT* genes between the genotypes in 0.5 ppm of P concentration, (**b**) relative fold change in expression of *OsPT* genes between the genotypes in 4.0 ppm of P concentration, (**c**) relative fold change in expression of *OsPT* genes between the genotypes in 10.0 ppm of P concentration, (**d**) relative fold change in expression of *OsPT* genes from 0.5 ppm to 4.0 ppm of P concentration, (**e**) relative fold change in expression of *OsPT* genes between 10.0 ppm and 4.0 ppm of P concentration, and (**f**) fold change in expression of *PSTOL1* between 0.5 ppm and 4.0 ppm and 100 ppm and 4.0 ppm of P concentration.

The expression of *PSTOL1* in IR64-Pup1 was considered as standard (unit value), and relative fold change in *PSTOL1* expression was calculated for Dular, IC459373, A.Kuruvai, and Sahbagidhan. ANOVA showed a significant difference (F-test, *p*-value: 0.014) between the genotypes for *PSTOL1* expression, but no significant difference was noticed when different P concentrations were considered (F-test, *p*-value: 0.775). Further, the expression of *PSTOL1* in IR64-Pup1 and Sahbagidhan was similar for all the P concentrations except for 0.5 ppm, which had a relatively low expression. However, *PSTOL1* expression was found to be significantly lower in A.Kuruvai than in IR64-Pup1 in all the P concentrations. Additionally, genotypes Dular and IC459373 showed relatively higher expression of *PSTOL1* than IR64-Pup1 in 0.5 and 10.0 ppm of P, while they were on a par in 4.0 ppm of P. The relative fold change in *PSTOL1* expression between the various P concentrations was also analyzed, considering the expression of *PSTOL1* in 4.0 ppm as standard (unit value). It was found that only two genotypes (IC459373 and Dular) showed relatively higher expression at 0.5 ppm of P than at 4.0 ppm (Fig. 7f). However, the expression was found to be similar for IR64-Pup1, Sahbagidhan, and A.Kuruvai in all three treatments. Additionally, *PSTOL1* was found to be unregulated at 10.0 ppm of P in Dular and IC459373.

### Expression analysis of phosphorus transporter genes

The expression of phosphorus transporter genes (*OsPT1* to *OsPT13*) in variety IR64 was kept as standard (unit value) and relative fold change in expression was studied in the remaining genotypes. There was no significant difference between either the genotypes or the transporter genes at 4.0 ppm of P concentration. In general, relative fold change expression among the genotypes was similar to that of IR64 except for Dular and IC459373, which showed relative downregulation of *OsPT2, OsPT5, OsPT6,* and *OsPT12* genes. However, there was significant upregulation of *OsPT8* only in genotype IC459373 (Fig. 7a–e). A similar analysis at 0.5 ppm of P concentration showed a significant difference (ANOVA, F-test: *p*-value: 0.015) only between the genotypes. Further, *OsPT1, OsPT2, OsPT6,* and *OsPT13* genes showed significant upregulation in Dular and IC459373 and *OsPT6* showed significant upregulation, specifically in IR64-Pup1. At 10 ppm of P concentration, upregulation of most of the *Os*PT genes occurred, and a significant difference was found between the genotypes as well as phosphorus transporter genes.

The relative fold change in *Os*PT gene expression between the treatments was also analyzed considering the expression of *Os*PT genes in 4.0 ppm of P as standard (unit value). The analysis showed the genes *OsPT1, OsPT2, OsPT8, OsPT10, OsPT12,* and *OsPT13* to have significant upregulation at 0.5 ppm of P vis-à-vis 4.0 ppm of P concentration (ANOVA, F-test: *p*-value: 0.010). Similarly, as compared with 4.0 ppm of P concentration, most of the genes showed upregulation at 10.0 ppm of P concentration, with a significant difference between the genotypes and genes (ANOVA, F-test: *p*-value: 0.005).

## Discussion

Phosphorus is an essential nutrient and is also a highly limiting factor in weathered tropical soils^18–20^. In plants, several P-responsive traits during different growth stages are well characterized. Our study showed high variability and morphological plasticity for most of the traits observed in rice under varying P concentrations. In agreement with our findings, reports by Luquet et al.^21^ also showed morphological plasticity in rice for low-P concentrations. However, experiments on the two-way interaction between genotypes and P concentrations (G × P) of previous reports were influential in understanding the P response in rice. Our report of three-way interaction (G × P × D) revealed that the P requirement of rice seedlings is higher during the early seedling stage (three-leaf stage/14 days) than in the four- to five-leaf stage (21‒28 days). The differential loading in PCA of the threshold 4.0 ppm concentration at 14 d and 28 d highlighted the importance of P in regulating root growth/vigor during the early growth stages in rice. This can be observed in Fig. 3 for the relationship between root dry weight and root parameters. Figure 3a illustrated that maximum root length had a strong association (83%) with root dry weight at 14 d and negligible association at 28 d (4%). In support of our findings, Julia et al.^22^ reported very early P uptake in 3-cm-long roots of rice even after only two days of the germination period. Strong expression of lipid-remodeling intolerant line Dular in the early stage (15 d) of rice plant growth under limited P suggests a higher requirement of P within the three- to four-leaf stage^23^. Additionally, the beneficial role of P during the early stages of rice growth and yield was reported by Ros et al.^24^ and Vandamme et al.^25^. In maize, P uptake during the one- to three-leaf stage (V1‒V3) regulates the growth of seedlings during the four- to six-leaf stage (V4‒V6) during seedling establishment^26^. In barley, P uptake from the second to fourth week is highly essential for grain yield^27^. Therefore, P uptake up to the three-leaf stage in rice is highly crucial for the establishment and yield of the rice crop.

**Figure 8:**
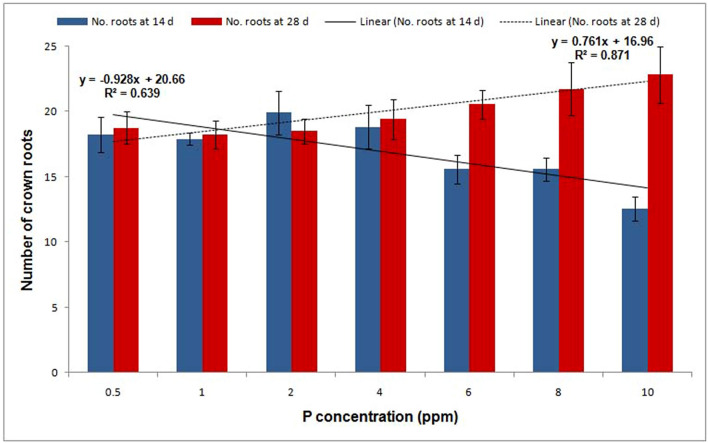
Average number of crown roots across genotypes under different concentrations of phosphorus in the hydroponic experiment at 14 d and 28 d. The vertical bar represents the standard deviation.

Phosphorus fertilization is essential for crop production due to widespread P deficiency throughout the world, along with soil P fixation^28, 29^. Practically, farmers used to apply fertilizer for rice only after the fifth- and third-leaf stages under transplanted and direct-seeded systems, respectively, which indicate less P availability invariably up to the three-leaf stage of growth. Furthermore, breeding strategies so far relied on either the number of tillers in low-P soil^30^ or root traits at later stages (five- to eight-leaf stage) for increasing phosphorus-use efficiency^31–33^. Thus, the genetic potential of genotypes for early root vigor (up to three-leaf stage) in low-P conditions for rice improvement is less explored so far in rice. In agreement with our observation, Julia et al.^22^ also suggested developing varieties with early root vigor for efficient P uptake. In this regard, we suggest different scenarios for consideration for the genetic improvement of PUE in transplanted and direct-seeded rice. In direct-seeded conditions, three-leaf-stage screening of genotypes/breeding lines for early seedling vigor can be adopted in addition to screening for tillers/yield in low-P fields. Moreover, major QTLs have been identified for lateral root numbers in direct-seeded rice^34^. However, the development of varieties with high root vigor is a big challenge for breeders^35^ because of the difficulty in screening for early root vigor under field conditions due to the delicate nature of seedlings that may result in a varying degree of root damage, thus causing a significant statistical error in root length measurements. The indirect selection of shoot traits highly correlated with early root length/root vigor could be highly useful in the hands of breeders for rice improvement^36, 37^. In support of this assumption, work on association mapping of seedling-related traits in rice showed a high correlation with shoot and root weight under normal conditions^36, 38^. In agreement with this finding, our analysis also showed a similar loading of shoot length with root and leaf length under low-P concentrations, indicating a high degree of correlation between these traits (Fig. 1a). Further, a 31% variation among the genotypes at 14 d was observed for shoot length, thus specifying the existence of genetic variation in rice cultivars. Thus, the selection of shoot length under contrasting P conditions will be able to identify lines/genotypes with early root vigor for varietal development. In our experiment, we observed that an increase in root length under low P had a significant effect on crown root number. We found that low P (0.5‒4.0 ppm) registered maximum root length with a lower number (18) of crown roots (Fig. 8), whereas 8.0‒10.0 ppm of P registered > 22 crown roots with decreased maximum root length (27.78%). Therefore, the trade-off between root length and crown root number should be considered when breeding for early root vigor in rice^39^.

**Figure 9:**
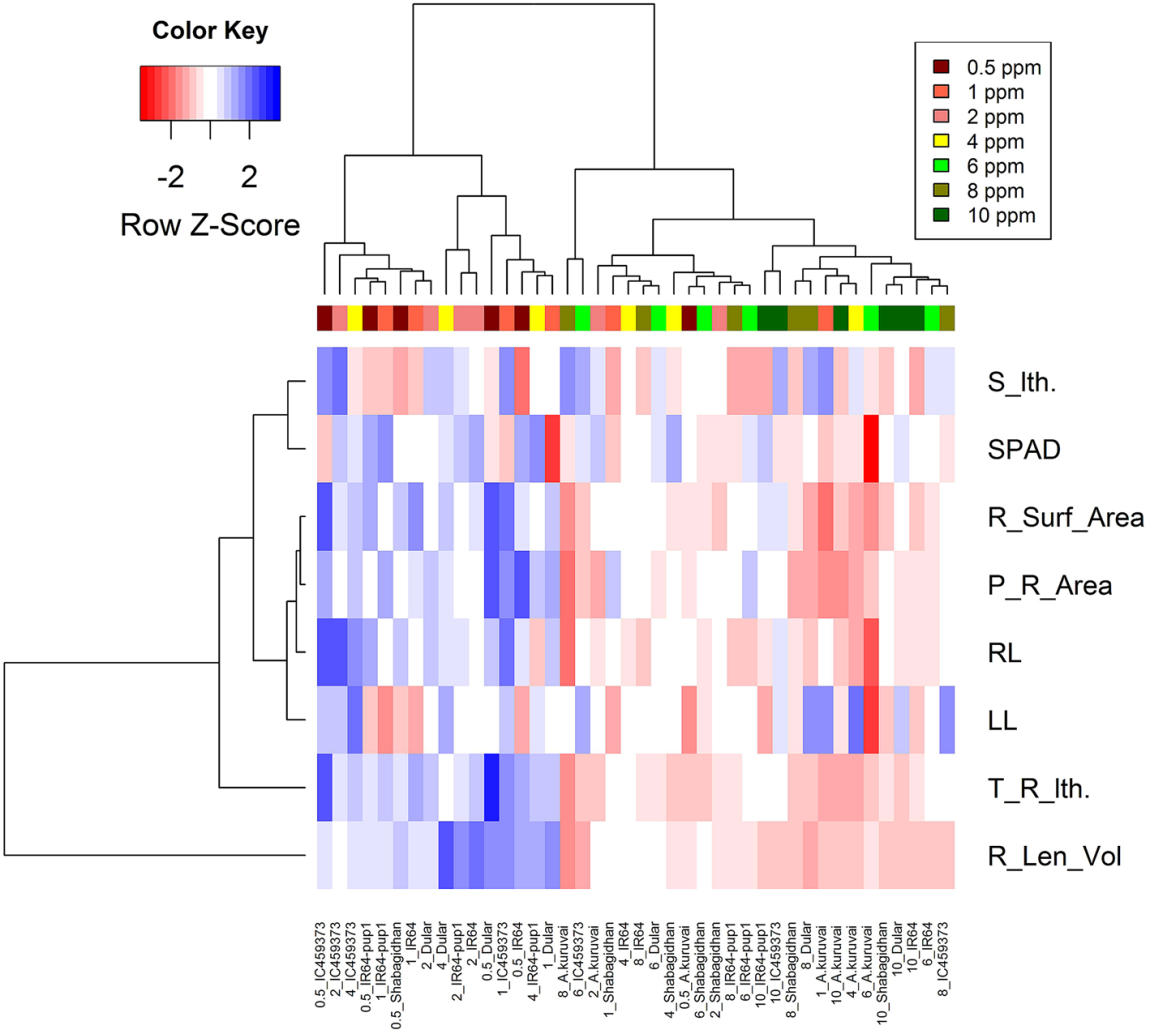
Heat map showing the grouping of genotypes based on similarity in expression of root and shoot vigor under different concentrations of P at 14 d. S_lth: shoot length (cm); SPAD; R_Surf_Area: root surface area (cm^2^); P_R_Area: projected root area (cm^2^); RL: max. root length (cm); LL: leaf length (cm); T_R_lth: total root length (cm); R_Len_Vol: root length per volume.

Rice is predominantly grown under transplanted conditions throughout the world. Further, early root vigor needs to be selected in the nursery for better performance under low-P conditions after transplanting of rice. In a previous report by Vandamme et al.^25^, two genotypes with low-P tolerance showed increased yield in the field due to enhanced seedling vigor upon the application of split doses of P in the nursery 1 week before sowing and another at 7 d after sowing. Thus, seedling vigor in the nursery contributes significantly to the yield of rice under transplanted conditions. In this regard, a similar selection strategy of evaluating shoot length in the nursery under contrasting P conditions up to the three-leaf stage would be able to identify lines for early root vigor. In addition, Li et al.^40^ reported that unique genomic regions were associated with low- and sufficient-P conditions, indicating that varied mechanisms exist in rice for different P concentrations. Therefore, the mapping and breeding approaches for early root vigor at the three-leaf stage in transplanted/direct-seeded rice would be to select breeding lines with relatively greater shoot length under low-P conditions and also for P responsiveness under P-sufficient field/nursery conditions. The previously characterized *PSTOL1* gene in rice provides tolerance of low-P conditions^14^. In agreement with these findings, genotypes IC459373, Dular, and IR64-Pup1 having *PSTOL1* genes showed high early root vigor in our analysis. However, for Sahbagidhan and A.Kuruvai, despite having a *PSTOL1* gene, their root vigor is not comparable to genotypes with high early root vigor. Further, heat map, correlation, and relative growth rate of early root vigor and shoot traits are high only in genotypes IC459373, Dular, and IR64-Pup1 (Fig. 9). Thus, donor selection with a functional *PSTOL1* gene is highly essential for a successful shoot length-based selection strategy for early root vigor in low-P conditions.

The identification and characterization of a novel source of tolerance of stresses are highly valuable because of the unique mechanisms that might be present in different tolerant donors^28^. For example, drought stress in rice increased the root-to-shoot ratio of the seedlings because of increased carbohydrate partitioning^41^. Therefore, characterizing genotypes with low-P tolerance and their further use in mapping populations require an efficient donor for early root vigor, as well as shoot vigor and any increase only in early root vigor, must not compromise overall seedling vigor. In our analysis, IC459373 with black hull color showed maximum root length with the maximum shoot and root dry weight, thus indicating efficient early root development.

From this study, we conclude that screening genotypes for low P at the three-leaf stage/14 days for shoot length would help in the identification of genotypes with early root vigor. However, screening genotypes directly for root traits using a high-resolution scanner would be useful to differentiate tolerant and susceptible genotypes at the early stage (14 d). This was well observed from the first principal component at 14 d and 28 d had a strong positive correlation with root length per volume. In addition, the number of pigmented leaves was strongly associated with root surface area, root length per volume, total root length, and the number of dry leaves under low P. The gain in size, weight, and diameter of roots under low P were accompanied by a ~ 2.5-fold rise in pigmented and senesced leaves. Decreased absorption of nutrients with an increase in root diameter could be the reason for enhanced dry-leaf numbers. IC459373 and Dular expressed an increased rate of growth for most of the root parameters under limited P, while the known drought-tolerant Sabhagidhan and short-duration genotype A.Kuruvai possessing *PSTOL1* had a slower rate of growth. *PSTOL1* had higher expression under low P and was also upregulated with an increase in the concentration of P. In addition to the presence of *PSTOL1* in IC459373 and Dular, the upregulation of *OsPT1*, *OsPT2*, *OsPT6*, and *OsPT13* could also contribute to P uptake under limited-P conditions. This suggests that amalgamation of *PSTOL1* and members of the *OsPHT1* gene family would be a prerequisite for improving tolerance and PUE in rice under limited-P conditions from the very early stage of seedling growth.

## Methods

### Plant materials

For the present experiment, six rice genotypes were evaluated in a series of concentrations of P in hydroponics. Three improved rice varieties, drought-tolerant rice genotype Sahbagidhan^42^, *PSTOL1* introgressed line IR64-Pup1 as a positive check^43^, and popular high-yielding rice variety IR64 as a negative check^44^, were used. These improved rice genotypes were developed by the International Rice Research Institute (IRRI), Philippines. The remaining three genotypes are landraces as Arupatham Kuruvai (A.Kuruvai) (AC 43847), Dular (IC 256827), and Kasalath (IC459373). A. Kuruvai and Dular were collected from National Rice Research Institute (NRRI), Cuttack, India and Kasalath was collected from the ICAR-National Bureau of Plant Genetic Resources, New Delhi, India. The rice genotypes were collected from respective institutes by following through the material transfer agreement (MTA), and further required compliance were taken from the institutes to execute the research work. The landraces of A.Kuruvai represent a very early maturing genotype, suitable for the direct-seeded and transplanted conditions of Tamil Nadu, India. A new indigenous accession with a black husk named Kasalath (IC459373) and a known donor with drought and low-P tolerance, Dular^45–47^, were used in this study. These genotypes underwent a PCR assay (data not shown) for the presence of the *PSTOL1* gene using established markers^47^. The seeds of all the genotypes were sorted for uniform seed size, and dormancy was broken by placing them in a hot-air oven at 50 °C for 45 h. Later, the seeds were surface-sterilized with 2.5% sodium hypochlorite for 20 min and washed five times to remove the traces of disinfecting agents using sterile water. For pre-germination, seeds were sown in a Petri dish on a paper towel moistened with distilled water for 72 h at 28 °C in an incubator.

### Experimental setup in screen-house conditions

The experiment was conducted in a net house located inside the campus of ICAR-NRRI, Cuttack (20° 27′ 09″ N, 85° 55′ 57″ E, 26 masl), in 2018. Healthy germinated seeds were meticulously transferred to a Styrofoam tray by guiding the roots using forceps. Styrofoam trays with seedlings were transferred to Yoshida nutrient solution^48^ with seven different concentrations: 0.5 ppm, 1.0 ppm, 2.0 ppm, 4.0 ppm, 6.0 ppm, 8.0 ppm, and 10.0 ppm of P. The NaH_2_PO_4_ served as a source of P in the nutrient solution, and different rates of concentration were used, as mentioned above, with three replications. Each rate of the P concentration was maintained in a separate dark plastic tray having a capacity of 10 L. All trays with seedlings received 13 h/11 h of day/night cycle, with an average temperature in the net house of 32 °C in the day and 23 °C at night, with an average light intensity of ~ 1200 µmol m^−2^ s^−1^ during the observation period. The seedlings were maintained for another 28 days, and data were observed on two instances (14 d and 28 d) after transferring the Styrofoam in hydroponics. The pH of the medium was preserved on alternate days from 4.50 to 4.55 and replaced every 7 d.

### Measurement of shoot and root component traits

**Figure 10:**
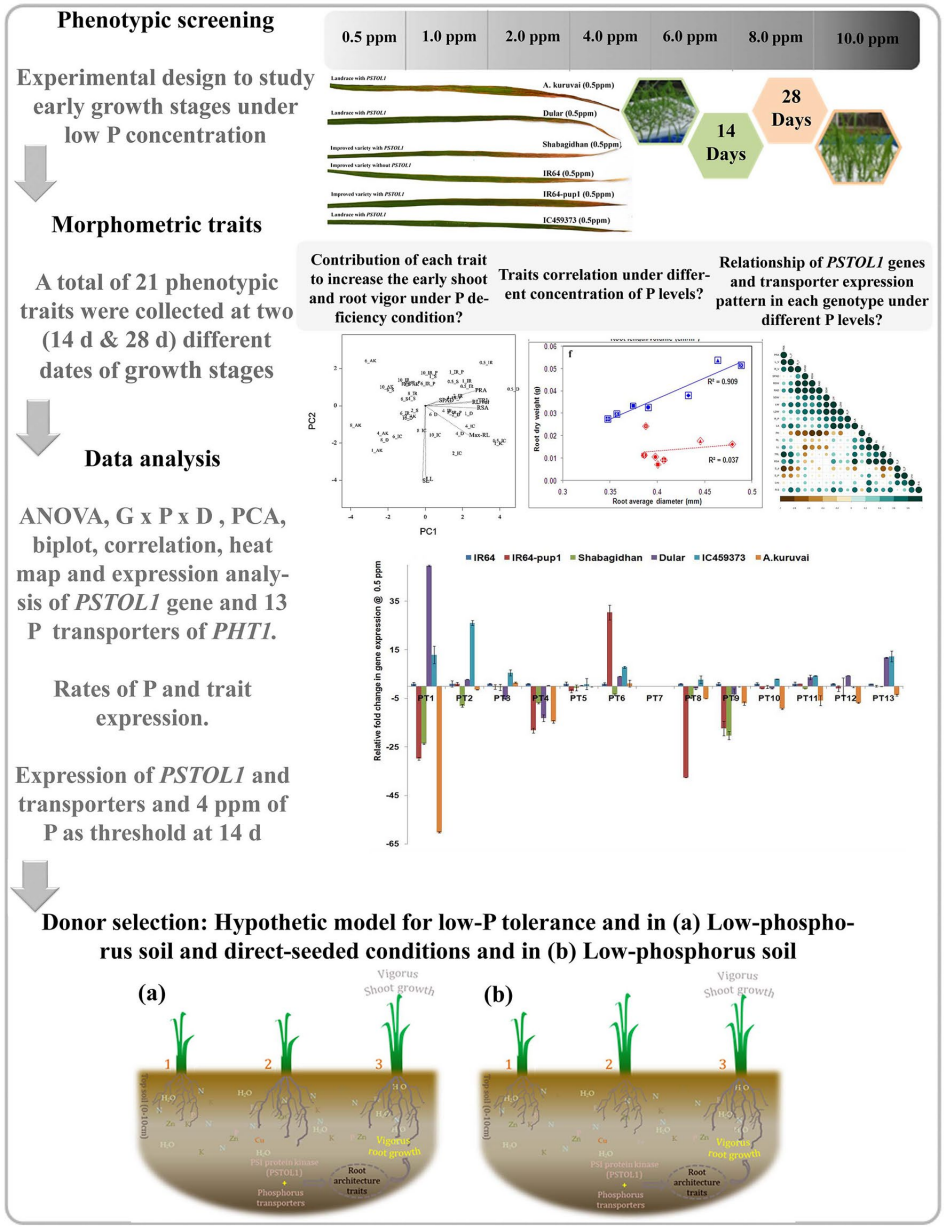
A schematic view of phenotypic screening under different concentrations of phosphorus and expression profiling of rice genotypes. Schematic representation of genotypes with the shoot and root vigor at 14 days (3‒4-leaf stage) after sowing in response to low P under direct-seeded (**a**) and nursery (**b**) conditions. (**a**) Low-phosphorus soil—young seedling under direct-seeded conditions: 1 = poor root and shoot vigor, 2 = root vigor but poor shoot growth, 3 = high root and shoot vigor. (**b**) Low-phosphorus soil—young seedling in the nursery: 1 = poor root and shoot vigor, 2 = poor root vigor but good shoot growth, 3 = high root and shoot vigor.

Data were collected on two occasions. Seedlings uprooted at 14 d and 28 d underwent measurement of 19 morphometric traits: shoot length (cm), maximum root length (cm), third-leaf length (cm), third-leaf width (cm), third-leaf area (cm^2^), number of leaves plant^−1^, crop nitrogen status using a chlorophyll meter (SPAD), stem thickness (mm), dry-leaf weight (g), stem dry weight (g), shoot dry weight (g), root dry weight (g), and root-to-shoot ratio. In contrast, root component traits such as total root length (cm), projected root area (cm^2^), root surface area (cm^2^), average root diameter (mm), root length per volume (cm/m^3^), and root volume (cm^3^) were measured by using commercial software package WinRHIZO Pro2013e (LA 2400, Regent Instrument Inc.) that works in Windows. All 19 traits were measured in both instances (14 d and 28 d) at all P rates. Also, tiller number plant^−1^, number of dry leaves plant^−1^, and pigmented leaves plant^−1^ (Fig. 10) were observed at 28 d in each treatment. Leaf greenness (chlorophyll) was measured using the SPAD (SPAD-502, Konica Minolta). The dry weight of shoot and root samples was determined when they were placed in a hot-air oven at 60 °C for 5‒6 days to estimate the shoot and root P content according to the method explained by^49^. The dried samples were milled into powder and stored in plastic bags at room temperature for P analysis. The samples were digested in a microwave oven followed by acid digestion by adding the concentration HNO_3_–HClO_4_ mixed acid (3:1). Inductively coupled plasma optical emission spectrometry (ICP-OES 710; Agilent Technologies, USA) was used to determine the content of P with the following parameters: radio frequency power, 1.1 KW; plasma gas flow, 15.0 L min^−1^; auxiliary gas flow, 1.50 L min^−1^; nebulizer pressure, 200 N; delay time, 15 s; flush time, 10 s; and read time, 5 s. Standard solutions of the eight mineral elements (1000 ppm, Merck, Germany) were used to obtain the calibration curve, with correlation coefficients of more than 0.999 in this study. Each sample was measured in triplicate. The total shoot and root P content was determined on an mg g^−1^ dry-weight basis.

To assess the influence of P on different component traits of shoots and roots, absolute or relative growth rate was calculated accordingly for all P rates and genotype-wise. Calculations were made as explained by^36^.

Absolute growth rate (AGR) was calculated as

$${\text{AGR}} = ({\text{A}}_{2} - {\text{A}}_{1} )/({\text{t}}_{2} - {\text{t}}_{1} )\;{\text{cm}}\;{\text{day}}^{{ - 1}}$$

where A_1_ and A_2_ are the length or width of the concerned trait measured at times t_1_ and t_2_, respectively.

Relative growth rate (RGR) was determined by using the dry weight of periodical observations and was represented as mg g^−1^ day^−1^.

$${\text{RGR}} = \left( {\log {\text{e}}\;{\text{W}}_{2} - \log {\text{e}}\;{\text{W}}_{1} } \right)/(t_{2} - t_{1} )$$

where W_1_ and W_2_ are the dry weight of the concerned trait at times t_1_ and t_2_, respectively.

Similar to the growth rate, the contribution of leaf/shoot/root systems to total plant weight was calculated for all P rates and genotype-wise. This was calculated as

$$\begin{aligned} & Contribution\;of\;roots/shoots/leaves\;in\;total\;plant\;dry\;weight\left( \% \right)~ \\ & \quad \quad = \frac{{Root/shoot/leaf\;dry\;weight}}{{Dry\;weight\;of\;roots\;and\;shoots}} \times ~100 \\ \end{aligned}$$

### RNA isolation and qPCR

The total RNA of root samples was isolated using the RNeasy kit, Quigen, Germany. Total RNA was isolated for three biological replicates of each sample and used for cDNA synthesis. The quality of the isolated RNA was analyzed in a nanodrop UV–visible spectrophotometer (Thermo Scientific, USA) and 1.5% agarose gel. Five µg of total RNA were treated with DNase I (NEB, USA) enzyme to remove the DNA contamination. Further, 2 µg of DNase I-treated RNA were used for first-strand cDNA synthesis using a prime script first-strand cDNA synthesis kit (Clontech). Quantitative-PCR for the expression of the phosphorus transporter (*OsPT*) genes was performed using a Mastercycler Realplex system (Eppendorf, Germany). Rice 18 s rRNA was used as a reference gene for cDNA normalization. For q-PCR analysis, three biological and technical replicates for each sample were used to identify the expression pattern of the *OsPT1 to OsPT13* genes (Table S2). SYBR, Premix Ex TaqII, Takara was used for the PCR cycle with the following cycle parameters: initial denaturation at 95 °C for 30 s, followed by 40 cycles of 95 °C for 5 s of denaturation and 60 °C for 30 s of annealing, and an extension cycle followed by melting curve analysis. The specificity of the amplicons was analyzed through the melting curve, and the ∆∆^CT^ method was used for fold-change expression analysis. The fold-change comparison was made by keeping the expression value of the gene in IR64 as one, and relative change in expression was calculated for other genotypes. Similarly, expression in 4 ppm was taken as one, and relative fold change in gene expression was calculated for 0.5 and 10.0 ppm.

### Statistical analysis

The morpho-physiological traits observed at 14 d and 28 d were analyzed by following 6 × 7 factorial RBD, while quantitative-PCR data from the experiments were analyzed by performing single factor analysis of variance (ANOVA) using Windostat 7.5 statistical computer software. Treatment differences across all P rates were estimated at the 1% and 5% levels of critical difference. Principal component analysis (PCA) was performed jointly for all the treatments with 21 traits at 14 d and 22 traits at 28 d to estimate the variability among genotypes and traits. Based on the variance estimated in PCA using all the attributes, a set of traits were selected. Those selected traits at both 14 d and 28 d had higher differences and then underwent PCA to narrow down the selection of highly variable traits that classify the P concentration and genotypes. Biplot figures explain the variances of the variables and correlation between the variables through vectors and the similarity between genotypes in the multivariate space based on the nature of the growth rate^50, 51^. PCA and biplot graphs were carried out between treatments (concentration) and traits across all P rates to understand the grouping pattern among the treatments, threshold, and responsiveness of the characteristics. Similarly, genotype vs. trait PCA and biplot was carried out to identify the responsiveness of the genotypes to different rates of P and the significant role of characteristics that differentiate the genotypes. These analyses were performed using Windostat 7.5 statistical computer software.

Linear regression was estimated between root dry weight and root component traits using MS Office Excel 2016. Pearson correlation analysis was performed for all P rates for both 14 d and 28 d using the corrplot functions from the corrplot package in R version (3.6.3)^52^. Traits registering a correlation value of ≥ 0.6 (*p* = 0.01 or 0.05) were considered for further discussion. A cluster heat map for all P rates at 14 d was prepared using a heat map.plus function (x, scale = “row”, dual scale = FALSE, method = “complete”) of the heat map.plus package^53^ by comparing the highly variable traits measured to identify the genotypes having high root and shoot vigor under low P (0.5‒4.0 ppm).

## Supplementary Information

Supplementary figure S1.

Supplementary figure S2.

Supplementary table S1.

Supplementary table S2.

## Data Availability

The datasets during or analyzed during the current study are available from the corresponding author upon request. The collection of the plant materials includes improved and wild rice varieties used in the present study complied with institutional, national, or international guidelines.
