# Supplementary material for: Trait variations and expression profiling of OsPHT1 gene family at the early growth-stages under phosphorus-limited conditions
Source: Sci Rep. 2021 Jun 30;11:13563. doi: 10.1038/s41598-021-92580-7 (PMC8245478; doi:10.1038/s41598-021-92580-7)
Supplement: Supplementary file 1 — Supplementary figure S1. [file 41598_2021_92580_MOESM1_ESM.pdf]

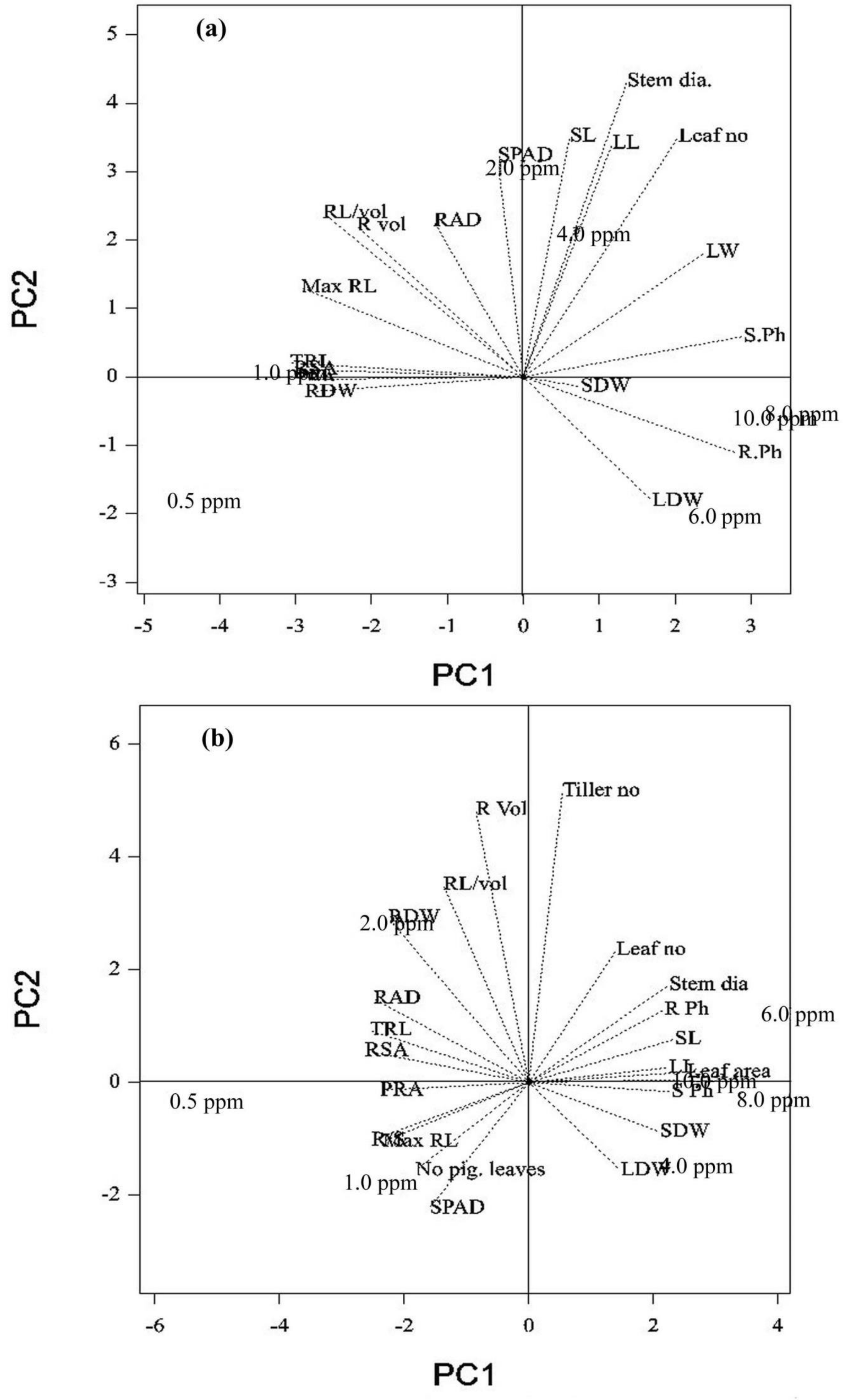

Figure S1. Concentration-by-trait biplot based on the variance exhibited by 21 traits at 14 days after sowing (DS) and 24 traits at 28 DS under different concentrations of phosphorus, explained by two principal component axes: (a) 14 DS and (b) 28 DS
