# Supplementary material for: Trait variations and expression profiling of OsPHT1 gene family at the early growth-stages under phosphorus-limited conditions
Source: Sci Rep. 2021 Jun 30;11:13563. doi: 10.1038/s41598-021-92580-7 (PMC8245478; doi:10.1038/s41598-021-92580-7)
Supplement: Supplementary file 2 — Supplementary figure S2. [file 41598_2021_92580_MOESM2_ESM.pdf]

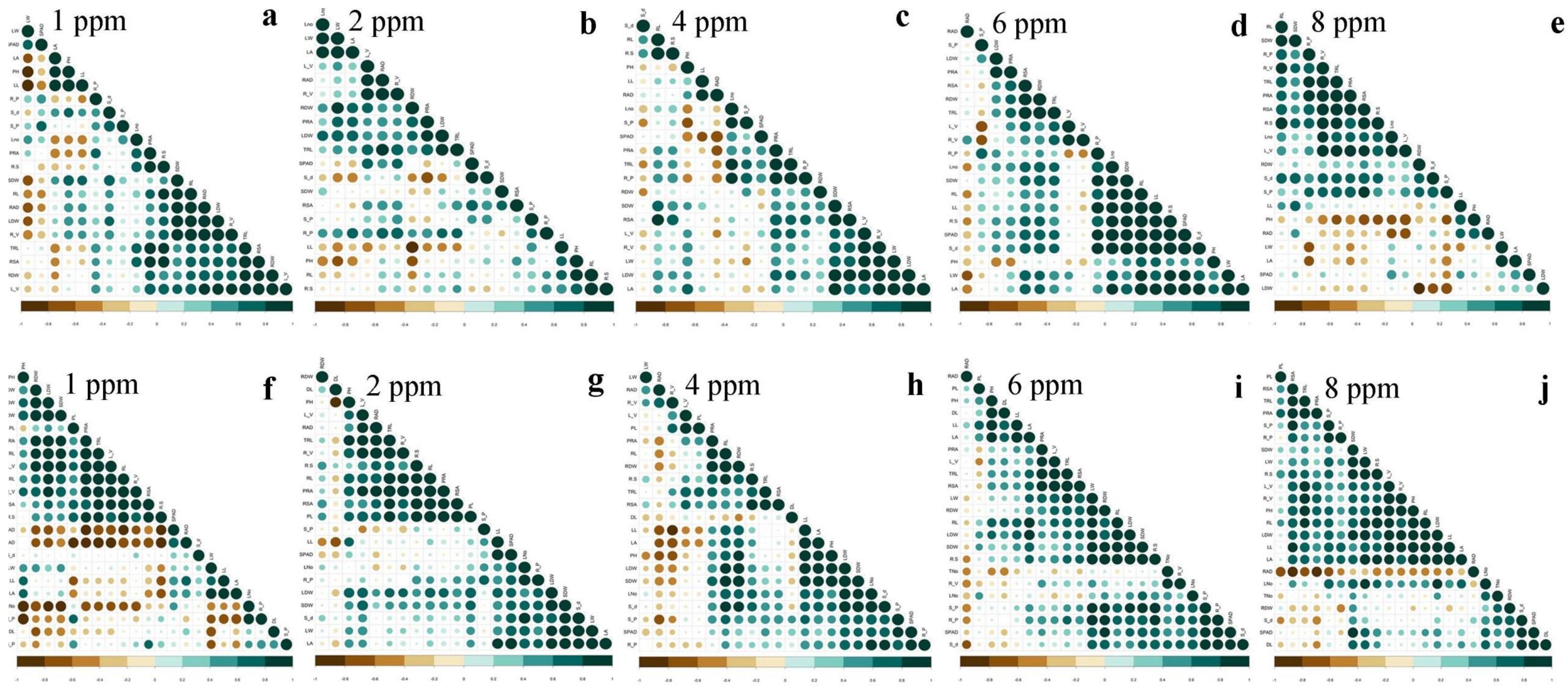

Figure S2. The correlation coefficient between morphological and physiological traits under varying concentrations of P (1.0–8.0 ppm). a-e: correlation coefficient at 14 d, f-j: correlation coefficient at 28 d. DL: number of dry leaves; L_V: root length per volume; LA: leaf area (cm^2); LDW: leaf dry weight (g); LL: leaf length (cm); LNo: number of leaves; LW: leaf width (cm); PH: shoot length (cm); PL: number of pigmented leaves; PRA: projected root area (cm^2); R.S: root-to-shoot ratio; R_P: root P content; R_V: root volume (cm^3); RAD: average root diameter (mm); RDW: root dry weight (g); RL: max. root length (cm); RSA: root surface area (cm^2); S_d: stem diameter (mm); S_P: shoot P content; SDW: shoot dry weight (g); SPAD; TRL: total root length (cm).
