# Supplementary material for: Trait variations and expression profiling of OsPHT1 gene family at the early growth-stages under phosphorus-limited conditions
Source: Sci Rep. 2021 Jun 30;11:13563. doi: 10.1038/s41598-021-92580-7 (PMC8245478; doi:10.1038/s41598-021-92580-7)
Supplement: Supplementary file 3 — Supplementary table S1. [file 41598_2021_92580_MOESM3_ESM.docx]

**Table S1.** Rate of growth and development of traits of *PSTOL1* possessing rice genotypes under different levels of P

| **Concentration** | **Genotype** | **Shoot length (cm day^-1^)** | **Root length (cm day^-1^)** | **3rd leaf length (cm day^-1^)** | **3rd leaf width (cm day^-1^)** | **Leaf area (mm day^-1^)** | **Leaf_no.** | **SPAD** | **Stem_diameter (mm day^-1^)** | **LDW (mg g^-1^day^-1^)** | **StDW (mg g^-1^day^-1^)** | **SDW (mg g^-1^day^-1^)** | **RDW (mg g^-1^day^-1^)** | **T_R_lth (cm day^-1^)** | **P_R_Area (cm^2^ day^-1^)** | **R_SurfArea(cm^2^ day^-1^)** | **R_Avg.Diam (mm day^-1^)** | **Len/Vol(cm/m^3^ day^-1^)** | **R_Vol(cm^3^ day^-1^)** | **S_P_conc (mg/g)** | **R_P_conc(mg/g)** |
| --- | --- | --- | --- | --- | --- | --- | --- | --- | --- | --- | --- | --- | --- | --- | --- | --- | --- | --- | --- | --- | --- |
| 0.5 ppm | Dular | 1.395 | 0.921 | 0.645 | 0.005 | 0.293 | 0.143 | 0.264 | 0.003 | 0.079 | 0.058 | 0.072 | 0.066 | 17.362 | 0.629 | 0.656 | 0.011 | 38.268 | 0.123 | 0.014 | 0.003 |
|  | Shabagidhan | 1.324 | 0.250 | 0.612 | 0.012 | 0.396 | 0.119 | 0.226 | 0.008 | 0.075 | 0.067 | 0.072 | 0.056 | 6.034 | 0.486 | 0.409 | 0.005 | 8.025 | 0.019 | 0.001 | 0.005 |
|  | IR64 | 1.364 | 0.438 | 0.610 | 0.007 | 0.417 | 0.143 | 0.140 | 0.003 | 0.070 | 0.067 | 0.069 | 0.044 | 5.822 | 0.149 | 0.398 | 0.001 | 10.477 | 0.020 | 0.003 | 0.004 |
|  | IR64-pup1 | 1.638 | 0.014 | 0.986 | 0.005 | 0.246 | 0.190 | 0.286 | 0.004 | 0.084 | 0.079 | 0.082 | 0.060 | 5.595 | 0.278 | 0.138 | 0.002 | 13.988 | 0.023 | 0.001 | 0.004 |
|  | A.kuruvai | 1.376 | 0.198 | 0.955 | 0.014 | 0.534 | 0.167 | 0.214 | 0.033 | 0.072 | 0.069 | 0.071 | 0.046 | 4.420 | 0.317 | 0.319 | 0.011 | 11.584 | 0.033 | 0.001 | 0.004 |
|  | IC459373 | 0.560 | 0.174 | 0.450 | 0.005 | 0.398 | 0.143 | 0.650 | 0.002 | 0.071 | 0.076 | 0.073 | 0.044 | 3.145 | 0.031 | 0.525 | 0.013 | 8.860 | 0.036 | 0.003 | 0.004 |
| 1 ppm | Dular | 1.852 | 0.802 | 0.483 | 0.014 | 0.667 | 0.095 | 0.705 | 0.035 | 0.158 | 0.122 | 0.147 | 0.076 | 17.398 | 1.060 | 0.622 | -0.016 | 34.594 | 0.174 | 0.011 | 0.003 |
|  | Shabagidhan | 1.276 | 0.288 | 0.712 | 0.005 | 0.454 | 0.095 | 0.521 | 0.042 | 0.085 | 0.074 | 0.081 | 0.079 | 4.628 | 0.048 | 0.394 | 0.008 | 15.028 | 0.031 | 0.004 | 0.004 |
|  | IR64 | 1.076 | 0.003 | 0.552 | 0.007 | 0.302 | 0.095 | 0.602 | 0.018 | 0.051 | 0.053 | 0.051 | 0.032 | 4.328 | 0.099 | 0.391 | -0.020 | 11.750 | 0.031 | 0.003 | 0.003 |
|  | IR64-pup1 | 1.390 | 0.069 | 0.850 | 0.017 | 0.160 | 0.143 | 0.657 | 0.025 | 0.091 | 0.084 | 0.088 | 0.078 | 2.366 | 0.305 | 0.423 | 0.008 | 12.084 | 0.026 | 0.002 | 0.004 |
|  | A.kuruvai | 1.002 | 0.207 | 0.333 | 0.005 | 0.306 | 0.119 | 0.262 | 0.020 | 0.061 | 0.062 | 0.061 | 0.043 | 2.365 | 0.007 | 0.150 | -0.004 | 11.965 | 0.011 | 0.003 | 0.005 |
|  | IC459373 | 1.007 | 0.119 | 0.900 | 0.010 | 0.407 | 0.143 | 0.207 | 0.031 | 0.075 | 0.076 | 0.076 | 0.054 | 2.096 | 0.334 | 0.334 | 0.004 | 11.075 | 0.021 | 0.002 | 0.003 |
| 2 ppm | Dular | 2.157 | 0.624 | 0.843 | 0.007 | 0.558 | 0.071 | 0.198 | 0.041 | 0.084 | 0.078 | 0.082 | 0.084 | 20.962 | 0.866 | 1.079 | 0.014 | 37.292 | 0.205 | 0.010 | 0.005 |
|  | Shabagidhan | 0.912 | 0.274 | 0.726 | 0.002 | 0.521 | 0.071 | 0.060 | 0.037 | 0.066 | 0.064 | 0.065 | 0.036 | 4.864 | 0.399 | 0.191 | 0.003 | 23.522 | 0.059 | 0.000 | 0.004 |
|  | IR64 | 0.681 | 0.005 | 0.300 | 0.000 | 0.255 | 0.071 | 0.140 | 0.041 | 0.040 | 0.043 | 0.041 | 0.128 | 4.636 | 0.191 | 0.706 | 0.007 | 19.238 | 0.033 | 0.004 | 0.004 |
|  | IR64-pup1 | 0.498 | 0.389 | 0.602 | 0.002 | 0.367 | 0.119 | 0.055 | 0.010 | 0.056 | 0.029 | 0.047 | 0.088 | 4.311 | 0.501 | 0.590 | -0.002 | 7.695 | 0.016 | 0.003 | 0.005 |
|  | A.kuruvai | 1.400 | 0.050 | 0.983 | 0.014 | 0.291 | 0.286 | 0.495 | 0.062 | 0.093 | 0.101 | 0.096 | 0.072 | 2.707 | 0.150 | 0.472 | -0.016 | 13.711 | 0.009 | 0.002 | 0.005 |
|  | IC459373 | 0.967 | 0.162 | 0.648 | 0.007 | 0.098 | 0.024 | 0.005 | 0.004 | 0.049 | 0.014 | 0.036 | 0.018 | 0.917 | 0.220 | 0.064 | 0.003 | 8.590 | 0.011 | 0.004 | 0.004 |
| 4 ppm | Dular | 1.421 | 0.245 | 0.657 | 0.000 | 0.697 | 0.071 | 0.095 | 0.030 | 0.061 | 0.075 | 0.066 | 0.095 | 3.892 | 0.553 | 0.215 | -0.005 | 13.790 | 0.001 | 0.011 | 0.004 |
|  | Shabagidhan | 1.200 | 0.155 | 1.071 | 0.017 | 0.445 | 0.095 | 0.164 | 0.003 | 0.068 | 0.075 | 0.070 | 0.076 | 3.325 | 0.314 | 0.450 | -0.004 | 15.934 | 0.012 | 0.007 | 0.004 |
|  | IR64 | 1.100 | 0.171 | 0.700 | 0.007 | 0.448 | 0.143 | 0.262 | 0.059 | 0.069 | 0.070 | 0.069 | 0.047 | 2.887 | 0.157 | 0.175 | 0.002 | 8.992 | 0.015 | 0.001 | 0.004 |
|  | IR64-pup1 | 1.095 | 0.143 | 0.629 | 0.010 | 0.454 | 0.071 | 0.093 | 0.045 | 0.062 | 0.071 | 0.065 | 0.050 | 2.088 | 0.114 | 0.387 | -0.002 | 12.380 | 0.008 | 0.003 | 0.004 |
|  | A.kuruvai | 1.286 | 0.024 | 0.538 | 0.014 | 0.200 | 0.000 | 0.200 | 0.008 | 0.056 | 0.061 | 0.058 | 0.039 | 1.464 | 0.226 | 0.051 | -0.005 | 11.775 | 0.009 | 0.001 | 0.004 |
|  | IC459373 | 2.810 | 0.198 | 2.126 | 0.010 | 0.284 | 0.190 | 0.343 | 0.071 | 0.109 | 0.107 | 0.109 | 0.090 | 1.135 | 0.261 | 0.068 | -0.009 | 7.267 | 0.002 | 0.004 | 0.004 |
| 6 ppm | Dular | 2.567 | 0.876 | 1.638 | 0.014 | 0.204 | 0.214 | 0.374 | 0.060 | 0.099 | 0.081 | 0.093 | 0.108 | 7.762 | 0.539 | 0.888 | -0.001 | 33.699 | 0.063 | 0.004 | 0.005 |
|  | Shabagidhan | 2.062 | 0.331 | 1.088 | 0.005 | 0.967 | 0.048 | 0.010 | 0.037 | 0.070 | 0.055 | 0.065 | 0.033 | 6.616 | 0.399 | 0.680 | -0.004 | 34.944 | 0.094 | 0.012 | 0.005 |
|  | IR64 | 1.136 | 0.221 | 0.814 | 0.014 | 0.975 | 0.119 | 0.395 | 0.058 | 0.072 | 0.064 | 0.069 | 0.057 | 4.275 | 0.414 | 0.423 | 0.000 | 26.546 | 0.031 | 0.001 | 0.004 |
|  | IR64-pup1 | 1.588 | 0.288 | 0.960 | 0.014 | 0.963 | 0.214 | 0.429 | 0.086 | 0.086 | 0.070 | 0.080 | 0.048 | 2.472 | 0.025 | 0.031 | -0.005 | 12.317 | 0.188 | 0.004 | 0.006 |
|  | A.kuruvai | 2.302 | 0.417 | 1.786 | 0.021 | 0.971 | 0.214 | 0.729 | 0.037 | 0.135 | 0.129 | 0.133 | 0.139 | 1.262 | 0.227 | 0.005 | -0.001 | 5.771 | 0.006 | 0.000 | 0.004 |
|  | IC459373 | 1.560 | 0.343 | 0.955 | 0.005 | 0.477 | 0.190 | 0.257 | 0.065 | 0.097 | 0.109 | 0.101 | 0.086 | 0.028 | 0.366 | 0.046 | -0.001 | 7.822 | 0.008 | 0.006 | 0.004 |
| 8 ppm | Dular | 1.357 | 0.333 | 0.479 | 0.012 | 0.632 | 0.119 | 0.124 | 0.043 | 0.062 | 0.073 | 0.065 | 0.144 | 6.967 | 0.752 | 0.675 | -0.004 | 20.210 | 0.022 | 0.012 | 0.004 |
|  | Shabagidhan | 2.431 | 0.481 | 1.067 | 0.026 | 0.301 | 0.214 | 0.352 | 0.018 | 0.124 | 0.076 | 0.106 | 0.020 | 6.778 | 0.363 | 0.590 | -0.004 | 27.086 | 0.026 | 0.003 | 0.006 |
|  | IR64 | 2.017 | 0.395 | 0.850 | 0.010 | 0.458 | 0.214 | 0.681 | 0.055 | 0.110 | 0.105 | 0.108 | 0.132 | 4.121 | 0.237 | 0.150 | 0.000 | 20.278 | 0.024 | 0.001 | 0.004 |
|  | IR64-pup1 | 0.464 | 0.143 | 0.436 | 0.007 | 0.882 | 0.071 | 0.140 | 0.048 | 0.055 | 0.048 | 0.053 | 0.082 | 2.183 | 0.489 | 0.007 | -0.002 | 10.167 | 0.011 | 0.001 | 0.005 |
|  | A.kuruvai | 1.588 | 0.188 | 0.864 | 0.021 | 0.260 | 0.262 | 0.400 | 0.052 | 0.090 | 0.089 | 0.090 | 0.123 | 2.159 | 0.164 | 0.344 | 0.000 | 11.838 | 0.012 | 0.003 | 0.006 |
|  | IC459373 | 1.650 | 0.321 | 0.781 | 0.007 | 0.593 | 0.190 | 0.629 | 0.046 | 0.072 | 0.093 | 0.080 | 0.126 | 2.018 | 0.385 | 0.094 | -0.002 | 9.601 | 0.010 | 0.005 | 0.004 |
| 10 ppm | Dular | 2.171 | 0.657 | 1.281 | 0.014 | 0.838 | 0.119 | 0.145 | 0.035 | 0.074 | 0.071 | 0.073 | 0.074 | 7.989 | 0.699 | 0.556 | -0.002 | 36.688 | 0.040 | 0.011 | 0.006 |
|  | Shabagidhan | 1.600 | 0.112 | 0.669 | 0.012 | 0.456 | 0.071 | 0.326 | 0.048 | 0.062 | 0.070 | 0.065 | 0.074 | 7.062 | 0.445 | 0.402 | 0.000 | 26.420 | 0.032 | 0.005 | 0.006 |
|  | IR64 | 0.626 | 0.155 | 0.483 | 0.005 | 0.450 | 0.095 | 0.071 | 0.003 | 0.034 | 0.050 | 0.040 | 0.128 | 5.099 | 0.462 | 0.409 | 0.000 | 28.557 | 0.034 | 0.000 | 0.006 |
|  | IR64-pup1 | 1.464 | 0.295 | 1.579 | 0.019 | 0.654 | 0.167 | 0.460 | 0.072 | 0.096 | 0.118 | 0.103 | 0.126 | 3.927 | 0.360 | 0.261 | 0.003 | 12.060 | 0.022 | 0.000 | 0.006 |
|  | A.kuruvai | 2.157 | 0.024 | 1.226 | 0.012 | 0.381 | 0.143 | 0.198 | 0.058 | 0.075 | 0.085 | 0.078 | 0.134 | 2.344 | 0.560 | 0.082 | -0.006 | 13.102 | 0.012 | 0.002 | 0.004 |
|  | IC459373 | 1.236 | 0.105 | 0.836 | 0.010 | 0.613 | 0.095 | 0.038 | 0.016 | 0.068 | 0.068 | 0.068 | 0.055 | 1.582 | 0.177 | 0.012 | -0.002 | 18.292 | 0.015 | 0.000 | 0.005 |
