# Supplementary material for: Trait variations and expression profiling of OsPHT1 gene family at the early growth-stages under phosphorus-limited conditions
Source: Sci Rep. 2021 Jun 30;11:13563. doi: 10.1038/s41598-021-92580-7 (PMC8245478; doi:10.1038/s41598-021-92580-7)
Supplement: Supplementary file 4 — Supplementary table S2. [file 41598_2021_92580_MOESM4_ESM.docx]

**Table S2** List of gene-specific primers used to amplify the *OsPHT1* family phosphate transporters *OsPT1* to *OsPT13* and *PSTOL1*

| **Sl No** | **Name** | **Sequence-F** (5’–3’) | **Sequence-R** (5’–3’) |
| --- | --- | --- | --- |
| 1 | OsPT1 | CGCTTCCGTACGAGTGGTAGT | GGTTCTTTCAAATCCAGGGAAA |
| 2 | *OsPT2* | AGCTGTTGGGTCGCCTTTACTACA | ACGACCATGAGGATGAGCGTGAAT |
| 3 | OsPT3 | TGCGACTGCTGATTCAGTACGT | ACAAATGCCATCAAATATGAACAGA |
| 4 | *OsPT4* | GGTTCACCGTCTTCTTCATCGACA | AGAAGGTGAAGGCGTACATGACCA |
| 5 | OsPT5 | TGCTACTGCCCATGACTAGGATT | CCATAGAAGAGATCCAGAGAAGCTGTA |
| 6 | *OsPT6* | ACGCTCTCAGGGCAACTCTTCTTT | TGAGCATGAGGGTGATGCCATAGA |
| 7 | *OsPT7* | TCCAAGGTGCTCCAGGTGAAGAT | TCTGGAACAGGTTCTGGGAGTAGT |
| 8 | *OsPT8* | TCCAGAAGGACATCTTCACCAGCA | ATGTCGATGAGGAAGACGGTGAAC |
| 9 | OsPT9 | AGAAAAACATAGGCTTGTCATCCTTT | AAAACCTAAGAAGCACTGTAAATAAATCC |
| 10 | OsPT10 | ATGTCGCCCATCCTTCCA | TCGCTTTCCGACGATGATC |
| 11 | *OsPT11* | AAGTTCAACGCGGCCAACAACTAC | AACATCTCCGTGAGAGCGTTGACT |
| 12 | *OsPT12* | TCATCGGAGCATTCGGTTTCCTCT | ATGTCATGAGGAACCCAACGAGGT |
| 13 | *OsPT13* | GGTGCCTTCATTGCTGCTGTCTTT | TGCTGTTTCTGGCATCTTCATGCG |
| 14 | *OsPSTOL1* | GTTTGTGGTGCATACAACTCGT | GGTTCCTCAAAAACAGAAGATG |
| 15 | OsACTIN | GGAACTGGTATGGTCAAGGC | AGTCTCATGGATACCCGCAG |
| 16 | *eEF-1α* | TTTCACTCTTGGTGTGAAGCAGAT | GACTTCCTTCACGATTTCATCGTAA |
